# A Genetic Screen Reveals an Unexpected Role for Yorkie Signaling in JAK/STAT-Dependent Hematopoietic Malignancies in *Drosophila melanogaster*

**DOI:** 10.1534/g3.117.044172

**Published:** 2017-06-15

**Authors:** Abigail M. Anderson, Alessandro A. Bailetti, Elizabeth Rodkin, Atish De, Erika A. Bach

**Affiliations:** Department of Biochemistry and Molecular Pharmacology, The Helen L. and Martin S. Kimmel Center for Stem Cell Biology, New York University School of Medicine, New York 10016

**Keywords:** *Drosophila melanogaster*, *hopscotch*, *Stat92E*, *hippo*, *expanded*, *yorkie*, *bantam*, *warts*, *Myc*

## Abstract

A gain-of-function mutation in the tyrosine kinase JAK2 (*JAK2^V617F^*) causes human myeloproliferative neoplasms (MPNs). These patients present with high numbers of myeloid lineage cells and have numerous complications. Since current MPN therapies are not curative, there is a need to find new regulators and targets of Janus kinase/Signal transducer and activator of transcription (JAK/STAT) signaling that may represent additional clinical interventions . *Drosophila melanogaster* offers a low complexity model to study MPNs as JAK/STAT signaling is simplified with only one JAK [Hopscotch (Hop)] and one STAT (Stat92E). *hop^Tumorous-lethal^* ^(^*^Tum-l^*^)^ is a gain-of-function mutation that causes dramatic expansion of myeloid cells, which then form lethal melanotic tumors. Through an F1 deficiency (Df) screen, we identified 11 suppressors and 35 enhancers of melanotic tumors in *hop^Tum-l^* animals. Dfs that uncover the Hippo (Hpo) pathway genes *expanded* (*ex*) and *warts* (*wts*) strongly enhanced the *hop^Tum-l^* tumor burden, as did mutations in *ex*, *wts*, and other Hpo pathway genes. Target genes of the Hpo pathway effector Yorkie (Yki) were significantly upregulated in *hop^Tum-l^* blood cells, indicating that Yki signaling was increased. Ectopic hematopoietic activation of Yki in otherwise wild-type animals increased hemocyte proliferation but did not induce melanotic tumors. However, hematopoietic depletion of Yki significantly reduced the *hop^Tum-l^* tumor burden, demonstrating that Yki is required for melanotic tumors in this background. These results support a model in which elevated Yki signaling increases the number of hemocytes, which become melanotic tumors as a result of elevated JAK/STAT signaling.

The JAK/STAT pathway is evolutionarily conserved and plays critical roles in numerous developmental processes, including hematopoiesis (Levy 1999; Amoyel *et al.* 2014). JAKs are nonreceptor cytosolic tyrosine kinases that are normally activated by cytokines interacting with their cell surface receptors. The activated JAK–receptor complexes induce phosphorylation of STATs that subsequently bind specific DNA sequences and act as transcription factors (O’Shea *et al.* 2002). A dominant-active allele *JAK2^V617F^* leads to a constitutively-active, ligand-independent protein (James *et al.* 2005; Jones *et al.* 2005; Kralovics *et al.* 2005; Levine *et al.* 2005). This mutation is a causal event in the development of the human MPNs polycythemia vera, essential thrombocythemia, and primary myelofibrosis (Tefferi 2016). In mouse models of MPNs, *JAK2^V617F^* ectopically activates STAT5, and genetic removal of *STAT5* impedes the development of these diseases (Walz *et al.* 2012; Yan *et al.* 2012; Sachs *et al.* 2016). MPN patients present with high numbers of myeloid lineage cells and a variety of symptoms, including splenomegaly, and they have complications such as heart attacks, stroke, and deep vein thrombosis (Wadleigh and Tefferi 2010). Multiple therapies including JAK2 inhibitors alleviate symptoms but are not curative (Tefferi 2016). Therefore, there is a pressing need to find new regulators and targets of JAK/STAT signaling that may provide druggable therapies.

In recent years, *Drosophila* has emerged as a powerful model organism for studying hematopoiesis [reviewed in Crozatier and Vincent (2011); Evans *et al.* (2014); Honti *et al.* (2014); and Gold and Bruckner (2015)]. There are two waves of hematopoiesis in *Drosophila*, one during embryogenesis and one during larval stages. The precursors of blood cells, called prohemocytes, form in the embryonic mesoderm, and most of them differentiate into plasmatocytes, which function as macrophages in removal of apoptotic corpses, wound healing, and immunity (Tepass *et al.* 1994; Wood and Jacinto 2007). Other prohemocytes develop into crystal cells, an insect-specific cell type that promotes melanization reactions (Lebestky *et al.* 2000). In larval stages, embryonically-derived plasmatocytes migrate to microenvironments called “hematopoietic pockets” located in the larval body wall (Makhijani *et al.* 2011; Makhijani and Bruckner 2012). In these segmentally-repeated pockets, plasmatocytes form resident (or sessile) clusters and are supported by the peripheral nervous system (Markus *et al.* 2009; Makhijani *et al.* 2011). Pocket-associated plasmatocytes self-renew and proliferate, resulting in a nearly 30-fold expansion of the plasmatocyte pool during larval development (Leitao and Sucena 2015; Petraki *et al.* 2015). At the earliest larval stages, all plasmatocytes reside in hematopoietic pockets. From the second larval instar, some plasmatocytes in the pockets are progressively released into circulation (Makhijani *et al.* 2011). However, certain conditions, like injury or immune challenge, cause sessile hemocytes to prematurely enter circulation in large numbers (Markus *et al.* 2009; Makhijani *et al.* 2011; Gold and Bruckner 2015). These pockets are sites of *bona fide* hematopoiesis because, in addition to promoting plasmatocyte self-renewal, resident plasmatocytes can differentiate into crystal cells (Bretscher *et al.* 2015; Leitao and Sucena 2015). Under immune challenge, like parasitoid wasp ovideposition, these resident cells can also differentiate into lamellocytes, which are large, flat, adherent cells that are normally absent in wild-type larvae (Markus *et al.* 2009). Lamellocytes encapsulate foreign objects such as parasitoid wasp eggs that are too large to be phagocytosed.

A second wave of hematopoiesis occurs in the larval lymph gland, a reservoir of prohemocytes that differentiate during second and third larval instars (Tepass *et al.* 1994; Lebestky *et al.* 2000; Mandal *et al.* 2004; Jung *et al.* 2005). Under normal, nonimmune challenged conditions, lymph gland prohemocytes give rise to ∼2000–3000 blood cells, most of which are plasmatocytes with a smaller number of crystal cells. Under immune-challenged conditions, lymph gland prohemocytes can also differentiate into lamellocytes (Rizki 1978; Evans *et al.* 2003; Jung *et al.* 2005). All hemocyte differentiation in the lymph gland is completed by early pupal development, and differentiated blood cells are released into circulation when this organ disintegrates (Grigorian *et al.* 2011). Lineage-tracing studies have shown that the hemocyte pool in the adult is comprised of cells from both the embryonic and larval lineages (Holz *et al.* 2003). It is currently debated whether there is hematopoiesis in the adult with most groups finding no evidence of *de novo* generation of blood cells at this stage (Rizki 1978; Lanot *et al.* 2001; Horn *et al.* 2014; Van De Bor *et al.* 2015).

The *Drosophila* JAK/STAT pathway is simplified with only three ligands, one cytokine receptor, one JAK (Hop), and one STAT (Stat92E) (Amoyel *et al.* 2014). A gain-of-function, temperature-sensitive mutation in the *Drosophila* JAK, *hop^Tum-l^*, causes a dramatic increase in plasmatocytes and lamellocytes, leading to the formation of melanotic tumors that are visible in larval, pupal, and adult stages (Figure 1B, arrow; Corwin and Hanratty 1976; Hanratty and Dearolf 1993; Luo *et al.* 1995, 1997). Prior reports have characterized the phenotype caused by the *hop^Tum-l^* mutation. Melanotic tumors are not observed in this genotype until the middle of the third larval instar (Hanratty and Ryerse 1981; Lanot *et al.* 2001). When reared at the restrictive temperature (29°), lymph glands from late second and early third instar *hop^Tum-l^* larvae lack the anterior lobe, the primary site of larval hematopoiesis in wild-type animals, but contain an increased number of hypertrophied secondary lobes. By late third instar at 29°, the lobes of the *hop^Tum-l^* lymph gland have dispersed, presumably releasing hemocytes into circulation (Hanratty and Ryerse 1981; Jung *et al.* 2005; Sorrentino *et al.* 2007). When reared at 25°, lamellocytes comprise 10–15% of circulating hemocytes in second instar *hop^Tum-l^* larvae, and this percentage increases to nearly 50% by third instar (Lanot *et al.* 2001). By contrast, lamellocytes are not observed in healthy wild-type animals reared under the same conditions. Additionally, the premature differentiation of lamellocytes in lymph glands of *hop^Tum-l^* larvae can be observed as early as the second instar (Lanot *et al.* 2001). Cells from *hop^Tum-l^* lymph glands are neoplastic and, when serially transplanted into naïve hosts, cause melanotic tumors for at least five generations (Hanratty and Ryerse 1981). Reducing the genetic dose of *Stat92E* markedly reduces the number and size of melanotic tumors in *hop^Tum-l^* animals (Luo *et al.* 1997; Shi *et al.* 2006).

**Figure 1:**
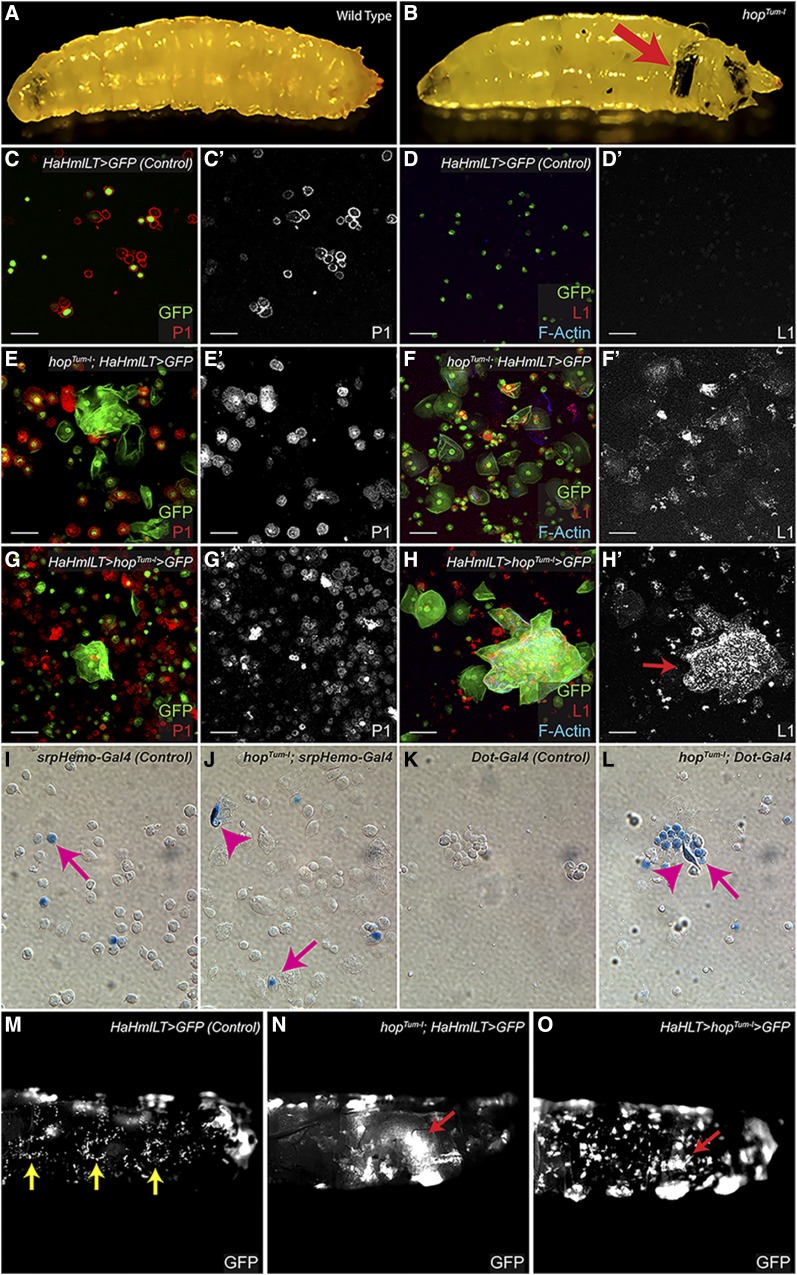
Characterization of the hematopoietic system in *hop^Tum-l^* animals. (A and B) Light micrographs of third instar wild-type (A) and *hop^Tum-l^* (B) larvae. The arrow in (B) highlights a large melanotic tumor in the *hop^Tum-l^* larva, which is not seen in wild-type (A). (C–H) Hemolymph bleeds from control *HaHmlLT* > *GFP* larvae reveal P1-positive (red) plasmatocytes (C) but not L1-positive (red) lamellocytes (D) in circulation. Hemolymph bleeds from *hop^Tum-l^*; *HaHmlLT* > *GFP* larvae reveal the presence of both P1- and L1-positive cells in circulation (E–F’). Bleeds from *HaHmlLT* > *hop^Tum-l^*, where *UAS-hop^Tum-l^* is mis-expressed in the entire hematopoietic compartment, show P1- and L1-positive cells in circulation (G–H’) as well as microtumors (H’, arrow). P1 is red in (C), (E), and (G); L1 is red in (D), (F), and (H). GFP labels hemocytes and F-actin is blue. (I–L) In control larvae, X-gal staining shows that *srpHemo-Gal4* lineage hemocytes (I, arrow) are present in larval circulation. In *hop^Tum-l^* larvae, X-gal staining reveals that the *srpHemo-Gal4* lineage gives rise to plasmatocytes and lamellocytes (J, arrow and arrowhead, respectively). In control larvae, no circulating cells are positive for the *Dot-Gal4* lineage (K). However, in *hop^Tum-l^* larvae, both plasmatocytes and lamellocytes arise from the *Dot-Gal4* lineage (L, arrow and arrowhead, respectively). (M–O) Hematopoietic pockets (M, arrows) from a control (*HaHmlLT* > *GFP*) larva where the entire hematopoietic compartment is labeled with GFP. Pockets are not observed in a *hop^Tum-l^*; *HaHmlLT* > *GFP* larva (N). The lack of hematopoietic pockets is autonomous to the hematopoietic lineage because hemocyte residence in these structures is not observed when *UAS-hop^Tum-l^* is mis-expressed in a *HaHmlLT* > *GFP* larva (O). The arrowheads in (N) and (O) mark GFP-positive microtumors within the abdominal cavity. Anterior is to the left and dorsal is up in (M–O). Bar, 50 μM. GFP, green fluorescent protein.

To identify genes that dominantly enhance or suppress the melanotic tumor burden in *hop^Tum-l^* animals, we conducted an F1 Df screen. Here, we report that genes encoding Hpo pathway components dominantly modify JAK/STAT-induced melanotic tumors. Hpo is a conserved tumor suppressor pathway that restrains the activity of Yki/Yes-associated protein (YAP) [reviewed in Irvine and Harvey (2015)]. Yki is a transcriptional coactivator that functions with several DNA-binding proteins, including a TEA/ATTS transcription factor Scalloped (Sd), to upregulate target genes that promote proliferation and inhibit apoptosis (Goulev *et al.* 2008; Wu *et al.* 2008). Through this F1 screen, we found that heterozygosity of *Df*(*2L*)*al* and of *Df*(*3R*)*ED6346* strongly enhanced the tumor burden in *hop^Tum-l^* animals. *Df*(*2L*)*al* uncovers *ex*, which encodes a protein that acts upstream of the core Hpo kinase cascade (Boedigheimer and Laughon 1993; Hamaratoglu *et al.* 2006), and *Df*(*3R*)*ED6346* uncovers *wts*, which encodes a serine–threonine kinase that inactivates Yki (Pantalacci *et al.* 2003; Udan *et al.* 2003; Wu *et al.* 2003). Mutations in *ex* and *wts* and in genes encoding other Hpo components significantly modified the tumor burden in *hop^Tum-l^* animals. Expression of the Yki target genes *bantam* (*ban*), a micro RNA (miRNA), and *Myc* (Thompson and Cohen 2006; Neto-Silva *et al.* 2010) were significantly increased in *hop^Tum-l^* hemocytes compared to controls, indicating that Yki has increased activity in *hop^Tum-l^* blood cells. Finally, we showed that Yki is necessary for JAK/STAT-dependent melanotic tumors as hematopoietic depletion of Yki from *hop^Tum-l^* animals significantly reduced the tumor burden. While ectopic activity of Yki in a wild-type background increased plasmatocyte proliferation, it did not induce lamellocyte differentiation or melanotic tumors. These results support a model where increased hematopoietic activity of Yki in *hop^Tum-l^* animals promotes proliferation of circulating plasmatocytes, which can be induced to transdifferentiate into lamellocytes by the ectopic JAK/STAT signaling in these cells.

## Materials and Methods

### Genetics and fly stocks

We obtained the following stocks from the Bloomington *Drosophila* Stock Center (BDSC): Df stocks covering chromosomes 2 and 3 from the Bloomington Deficiency Kit (Cook *et al.* 2012); *Oregon* (*Ore*)*^R^*; *hop^Tum-l^* (FBgn0004864); *ex^e1^*, *ex^NY1^*, *ex^697^* (FBgn0004583); *hippo* (*hpo*)*^KC202^*, *hpo^42-47^* (FBgn0261456); *Stat92E^397^* (FBgn0016917), a strong hypomorphic allele (Ekas *et al.* 2010); *mob as tumor suppressor* (*mats*)*^12-4^* (FBgn0038965); *salvador* (*sav*)*^3^* (FBgn0053193); *kibra^02404^* (FBgn0262127); *crumbs* (*crb*)*^j1B5^*, *crb^07270^* (FBgn0259685); *fat* (*ft*)*^8^* (FBgn0001075); *discs overgrown* (*dco*)*^j3B9^* (FBgn0002413); *echinoid* (*ed*)*^K01102^*, *ed^KG04279^* (FBgn0000547); *wts^X1^* (FBgn0011739); *yki^B5^* (FBgn0034970); *Tub-EGFP.ban* (also known as the *ban* sensor); *UAS-yki^S168A.V5^*. For Yki RNAi, we used TRiP lines *HMS00041* and *JF03119* (BL-34067 and BL-31965, respectively). We used *thread ^j5c8^* (FBgn0260635) (also called *Diap1-lacZ*, gift from H.D. Ryoo, NYU); *Hemolectin* (*Hml*)Δ*DsRed* [gift from K. Brückner, UCSF (Makhijani *et al.* 2011)]; *Hml*Δ*-Gal4*; *UAS-hop^Tum-l^* (see below); *Hand-gal4*, *Hml*Δ*-Gal4*, *UAS-FLP.JD1*, *UAS-2xEGFP*; *Gal4-Act5C* (*FRT.CD2*) [referred to as *HaHmlLT* > *GFP*, a gift from U. Banerjee, UCLA (Mondal *et al.* 2014)]. We derived *Hand-Gal4*, *Hml*Δ*-Gal4*, *UAS-2xEGFP* (termed *HaHml-Gal4* > *GFP*) from the *HaHmlLT-Gal4* line. The Gal4/UAS system (Brand and Perrimon 1993) was used for mis-expression and RNAi. Unless specified, all crosses were reared at 25°. For the lineage-tracing, we used *Dorothy* (*Dot*)-*Gal4* (a gift of U. Banerjee) and *srpHemoGal4*, *UAS-srcEGFP* (Bruckner *et al.* 2004) and this flip-out stock: *y w UAS-flp*; *tub-Gal80^ts^*; *act5c FRT stop FRT nuc lacZ/Tb* (Makhijani *et al.* 2011), the latter two gifts of K. Brückner. *HaHmlLT-Gal4* and its derivative *HaHml-Gal4*, have broader expression patterns than *Hml*Δ*-Gal4*, as the former two are expressed in lymph gland progenitors and other cell populations (Mondal *et al.* 2014). *Hml*Δ*-Gal4* is restricted to embryonic-lineage plasmatocytes and, later in larval development, to lymph gland hemocytes (Goto *et al.* 2003; Jung *et al.* 2005).

### UAS-hop^Tum-l^ transgenic line

The *UAS-hop^Tum-l^* plasmid (Harrison *et al.* 1995) was a gift from R. Binari and N. Perrimon, Harvard Medical School. This plasmid was injected into *w^1118^* embryos by BestGene Inc. (Chino Hills, CA). One line *UAS-hop^Tum-l^* 2M is a stable insertion on X that is hemi- and homozygous viable. When mis-expressed in hematopoietic tissue, this line induced melanotic tumors. This line was used for all *UAS-hop^Tum-l^* mis-expression experiments.

### Screen design and scoring methods

*hop^Tum-l^* is an allele on the X chromosome marked by a mutation in *vermilion* (*v*). To remove second site mutations that may have accumulated on the *v*, *hop^Tum-l^* chromosome, we employed meiotic recombination in females by crossing *v*, *hop^Tum-l^* virgins to isogenized *Ore^R^* males. We isolated single female recombinants from this cross that were mutant for *v* and had melanotic tumors (and therefore had retained the *hop^Tum-l^* allele). We selected one line (#13) for the screen that had an increase in the number and size of melanotic tumors compared to the parental line. For the screen *v*, *hop^Tum-l^*/*FM7* #13 virgin females (hereafter referred to as *hop^Tum-l^* females) were crossed to males from the Bloomington Df Kit. For each batch of Dfs that we tested, we set up (1) a cross of *hop^Tum-l^*/*FM7* virgins to *Ore^R^* males, the progeny of which were used for normalization of the tumor index (TI, see below), and (2) a cross of *hop^Tum-l^*/*FM7* virgins to *Stat92E^397^/TM6B*, *Tb* males, the progeny of which were used to mark suppression of the tumor phenotype. Melanotic tumors in F1 adult progeny were scored as quarters of adult abdominal segments using a dissecting microscope. For example, a tumor that covers one quarter of a segment was scored as 0.25, a tumor covering one half of a segment was scored as 0.5, and a tumor that covers one complete abdominal segment was classified as a 1.0, and so forth. Each individual progeny was given a TI, which is the sum of all tumor sizes per animal [adapted from Shi *et al.* (2006)]. For statistical purposes, the TI for each individual was normalized to the average TI of the control *hop^Tum-l^*/+ outcross set up in parallel and divided by the SD of that control using the following formula: [Tl]_normalized_ = ([TI]_Df_ – [TI]_control_)/[SD]_control_. We then computed the average normalized TI (NTI) of all individuals in each genotype. NTIs were graphed with SE bars by GraphPad Prism 7. Genotypes that significantly modified the NTI by 1 SD above or below that of the control were classified as enhancers or suppressors, respectively. Except for rare cases of unhealthy crosses, the minimum sample size of each Df screened (*hop^Tum-l^/+*; *Df/+*) was 15. Modifying Dfs were independently validated.

### Immunohistochemistry

Hemocytes were bled from wandering third instar larvae into 30 μl phosphate buffered saline (1× PBS) and allowed to settle for 30–45 min on Superfrost Plus microscope slides (Fisher Scientific Catalog# 1255034). Hemocytes were fixed with 4% formaldehyde or 4% paraformaldehyde in PBS for 10 min, washed twice for 10 min in 0.1% Triton, 1× PBS (PBS-T), and then blocked for 1 hr at room temperature or overnight at 4° in 10% normal goat serum (NGS, Vector, Catalog# S-1000) in PBS-T. Hemocytes were incubated in primary antibody diluted in blocking solution overnight at 4°, with the exception of anti-Stat92E, which was incubated overnight at room temperature. The following antibodies were used: rabbit anti-Stat92E [1:250, Flaherty *et al.* (2010)]; rabbit anti-Myc (1:1000, a gift of R. Eisenman); mouse anti-P1 (Nimrod C) and mouse anti-L1 (Atilla) [both at 1:10, a gift of I. Andó (Kurucz *et al.* 2007)], mouse anti-βGal (1:250, Developmental Studies Hybridoma Bank); chicken anti-βGal (1:250, Immunology Consultants Lab, Catalog# CGAL-45A-Z); chicken anti-GFP (1:500, Aves labs, Catalog# GFP-1020); rabbit anti-GFP (1:500, Molecular Probes Catalog# A6455); rabbit anti-RFP (1:500, Medical & Biological Laboratories Co., Ltd., Catalog # PM005); guinea pig anti-Scalloped [Sd, 1:500, gift from J. Zeitlinger (Ikmi *et al.* 2014)]; mouse anti-LaminD (1:100); and Phalloidin, which stains F-actin (1:25, Molecular Probes). All secondary antibodies (Jackson Immunologicals) were used at 1:200. Samples were mounted in Vectashield (Vector, Catalog# H-1000). Images were acquired using a Zeiss LSM510 confocal microscope. Images were processed using Adobe Photoshop and figures were made with Adobe Illustrator.

For mixed cell experiments, hemolymph from DsRed-positive control larvae was bled into the same well as that from unlabeled experimental larvae. Fixation and immunostaining were performed as described above.

### 5-ethynyl-2′-deoxyuridine (EdU) labeling

EdU-labeling of hemocytes was performed using the Click-iT EdU Alexa Fluor 647 Imaging Kit (Invitrogen, Molecular Probes #C10356). Hemocytes from third instar larvae were dissected into 15 μl of 10 μM EdU and allowed to settle for 30 min into 5 mm wells on Superfrost Plus microscope slides made with a pap pen (Sigma-Aldrich #Z377821). After 30 min of EdU incorporation, the solution was removed and replaced with 4% paraformaldehyde for 10 min. The samples were washed twice with PBS-T. Samples were incubated with primary mouse anti-P1 and fluorescent secondary antibodies as described above. The cycloaddition reaction was performed per the manufacturer’s instructions. Samples were mounted in Vectashield. We imaged 10–15 areas of the slide at random at 25× using a Zeiss LSM510 confocal microscope. To calculate the percentage of proliferating plasmatocytes, we used ImageJ to quantify the number of P1-positive and EdU-positive cells.

### Statistics

For quantification of intensity of GFP, Myc, or Stat92E, we used ImageJ. First, we defined single cells (either DsRed-positive control hemocytes or DsRed-negative *hop^Tum-l^* (or *Hml* > *Yki^S168A.V5^*) hemocytes) in a single 1 μm confocal slice or maximum intensity projection. We then determined the average gray value of the green channel for each genotype. The gray values of the controls were normalized to 1, and then the *hop^Tum-l^* samples were normalized to the control.

All numerical data were plotted and analyzed using GraphPad Prism7, and Student’s *t*-tests were used determine statistical significance.

### Lineage-tracing experiments

*y w UAS-flp/Y*; *tub-Gal80^ts^*; *act5c FRT stop FRT nuc lacZ/Tb* males were crossed separately to virgins of these four genotypes: (1) *srpHemoGal4*, *UAS-srceGFP*; (2) *hop^Tum-l^/FM7*; *srpHemoGal4*, *UAS-srceGFP*; (*3*) *Dot-Gal4*, and (4) *hop^Tum-l^/FM7*; *Dot-Gal4*. Crosses were reared at 29° to inactivate Gal80. Hemocytes were bled from wandering third instar larvae into 30 μl 1× PBS and allowed to settle for 30 min on Superfrost Plus microscope slides. They were then fixed in 1% glutaraldehyde for 15 min, followed by X-gal staining at 37° for 30 min. DIC images of hemocytes (at 40×) were obtained using a Zeiss Axioplan microscope with a Retiga Evi (QImaging) digital camera and QCapture Pro 6.0 software.

### Hematopoietic pockets

*HaHmlLT* > *GFP* flies were crossed with *Ore^R^*, *UAS-hop^Tum-l^*, *hop^Tum-l^*/*FM7*, or *UAS-Yki^S168A.V5^* flies and reared at 29°. Third instar larvae of the correct genotype were collected and washed in 1× PBS. Handling of the larvae, which can disrupt hematopoietic pockets (Makhijani *et al.* 2011), was kept to a minimum. Larvae were immobilized on chilled microscope slides and images were obtained using a Nikon D5100 camera mounted on a Nikon SMZ 1500 dissecting microscope with UV X-Cite 120 at 5× magnification.

### Data availability

Stocks are available upon request or from the BDSC. Table S1 contains the full data set for the *hop^Tum-l^* Df screen. Figure S1 contains expression patterns of *Diap1-lacZ* and Scalloped in control and *hop^Tum-l^* hemocytes. Figure S2 contains expression pattern of Stat92E in in control and *hop^Tum-l^* hemocytes.

## Results and Discussion

### Characterizing the hop^Tum-l^ allele

After cleaning up the *hop^Tum-l^* chromosome (see *Materials and Methods*), we wanted to characterize this allele. We crossed virgin *hop^Tum-l^* females balanced over an *FM7* chromosome to *Ore^R^* males. At 25°, 68% of heterozygous *hop^Tum-l^/+* adult animals developed melanotic tumors. At 29°, the penetrance increased to 99%, consistent with an earlier report (Corwin and Hanratty 1976). We labeled the entire hematopoietic lineage using the *HaHmlLT-Gal4* driver and *UAS-GFP* crossed to Ore^R^ (for the control) or *hop^Tum-l^*. As expected, hemolymph bleeds from control *HaHmlLT* > *GFP* larvae contained P1-positive plasmatocytes but no lamellocytes (Figure 1, C–D’). By contrast, hemolymph bleeds from *hop^Tum-l^*; *HaHmlLT* > *GFP* third instar larvae revealed the presence of both plasmatocytes and lamellocytes [Figure 1, E–F’ and Lanot *et al.* (2001)]. The precocious appearance of lamellocytes in *hop^Tum-l^* animals is due to autonomous activation of the JAK/STAT pathway within hemocytes because these observations can be recapitulated by mis-expression of *UAS-hop^Tum-l^* within the hematopoietic lineage (Figure 1, G–H’). Because the lymph gland in *hop^Tum-l^* animals disperses prior to pupariation, we wanted to determine whether lymph gland-derived hemocytes were present in the circulating pool in *hop^Tum-l^* larvae (Hanratty and Ryerse 1981; Jung *et al.* 2005; Sorrentino *et al.* 2007). We used a lineage-tracing technique and Gal4 lines that are restricted to the embryonic lineage [*srpHemo-Gal4* (Bruckner *et al.* 2004)] or larval lymph gland [*Dot-Gal4* (Honti *et al.* 2010)]. As expected, we found embryonic-lineage hemocytes in larval circulation in controls [Figure 1I, arrow and (Bruckner *et al.* 2004)]. In *hop^Tum-l^* larvae, embryonic-lineage hemocytes gave rise to labeled plasmatocytes as well as lamellocytes (Figure 1J, arrow and arrowhead, respectively). In control larvae, lymph gland-lineage cells were not found in circulation (Figure 1K). In contrast, lymph gland-lineage hemocytes, including cells with lamellocyte characteristics, were present in circulation in *hop^Tum-l^* larvae (Figure 1L, arrow and arrowhead). At larval stages, sessile hemocytes reside in hematopoietic pockets in the body wall (Markus *et al.* 2009; Makhijani *et al.* 2011). Whereas hematopoietic pockets were apparent in control *HaHmlLT* > *GFP* larvae (Figure 1M, arrows), they were not observed in *hop^Tum-l^*, *HaHmlLT* > *GFP* larvae (Figure 1N). The cell-autonomy and specificity of this result were confirmed by mis-expressing *UAS-hop^Tum-l^* using the *HaHmlLT-Gal4* driver. No pockets were observed in *HaHmlLT* > *hop^Tum-l^* larvae (Figure 1O). Therefore, in *hop^Tum-l^* larvae the circulating pool of hemocytes is comprised of both embryonic- and lymph gland-derived cells, and there are alterations in hemocyte residence in larval hematopoietic pockets.

### A screen for modifiers of hop^Tum-l^ melanotic tumors

**Figure 2:**
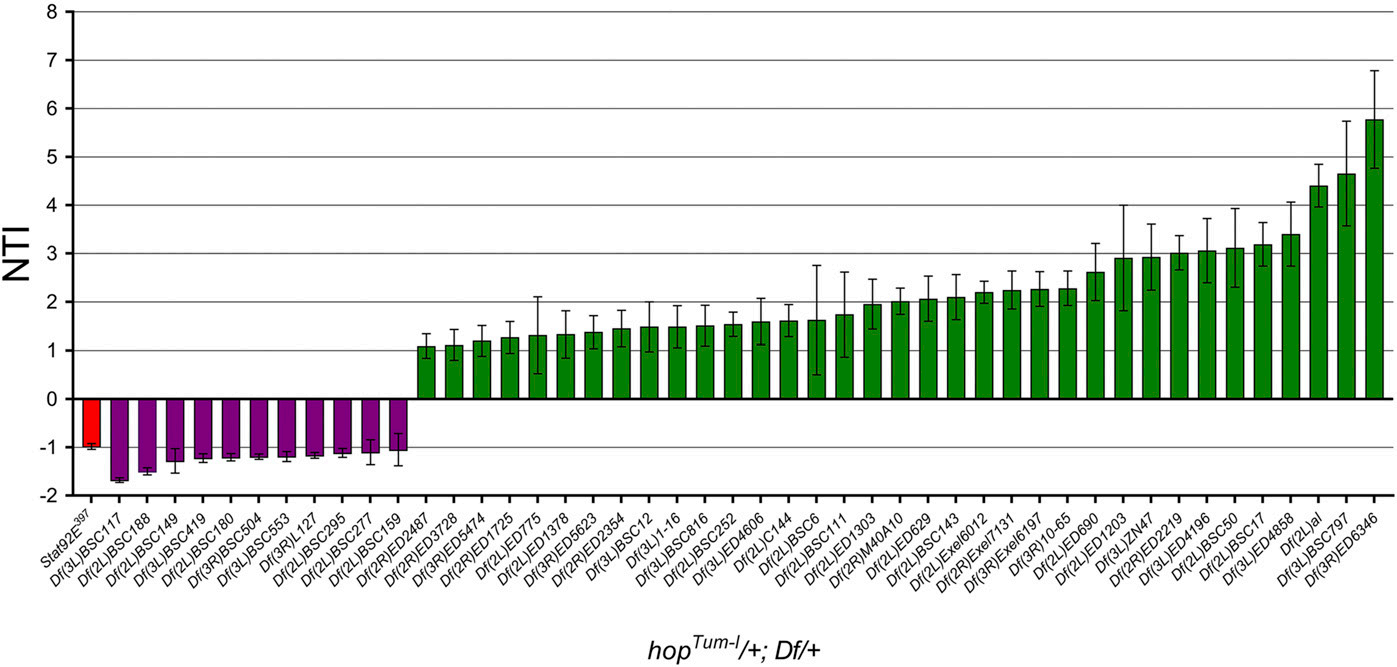
An F1 screen identified 35 Dfs that enhance and 11 that suppress *hop^Tum-l^* melanotic tumors. We tested 363 stocks from the Bloomington Df Kit. We crossed males from each Df to *hop^Tum-l^* virgin females. F1 adult progeny (*hop^Tum-l^*/+; Df/+) were scored for the number and size of abdominal melanotic tumors as described in the *Materials and Methods*. The graph shows the NTI ± SE on the *y*-axis for each Df that was significant [*i.e.*, NTI >1 SD above or below the NTI for the control (*hop^Tum-l^*/+; +/+; +/+), which was set at 0]. The names of the Dfs are indicated on the *x*-axis. The red bar indicates the positive control for suppression, heterozygosity for the strong hypomorphic allele *Stat92E^397^*. The purple bars indicate suppressors and the green bars indicate enhancers. Df, deficiency screen; NTI, normalized tumor index.

We wanted to establish an F1 genetic screen with the goal of identifying novel modifiers of JAK/STAT-induced melanotic tumors. We first screened the left arm of the second chromosome (2L). *hop^Tum-l^* virgin females were crossed to Df males, and the crosses were reared at 29°. F1 adult females that were heterozygous for *hop^Tum-l^* and for the Df were scored for the presence and size of abdominal melanotic tumors. As described in the *Materials and Methods*, we used the abdominal segments as a ruler to measure how much of each segment a melanotic tumor covered. The sum of all tumor sizes within an adult was that individual's TI. A normalized TI (NTI) for each individual adult was obtained by comparing its TI to that of an outcross control (*hop^Tum-l^/FM7* virgins × *Ore^R^* males) set up at the same time and reared under identical conditions. The NTI for each genotype was calculated by averaging all of the TI scores for each individual in that genotype. An interaction was considered significant if the NTI was > 1 SD above or below the relevant control (see *Materials and Methods*). Although we observed increased lethality accompanied by lower recovery of progeny at 29°, we identified multiple Dfs on 2L that significantly modified the *hop^Tum-l^* melanotic tumor phenotype (Figure 2 and Table S1). However, we chose to perform the rest of screen at 25° to mitigate the enhanced lethality at 29°.

Overall, we tested 363 fly stocks, covering chromosomes 2 and 3, from the Bloomington Df Kit. The majority of Dfs tested fell within 1 SD of the control and were considered to have no effect (see *Materials and Methods*, Table S1). Two Dfs resulted in synthetic lethality as no adult offspring were recovered (Table S1). However, 46 Dfs significantly altered the *hop^Tum-l^* melanotic tumor phenotype (Figure 2 and Table S1). Specifically, 35 Dfs enhanced the NTI, while 11 Dfs suppressed it. We compared our list of modifier regions to genes previously reported to be involved in inducing blood cell masses when mutated or overexpressed (Betz *et al.* 2001; Hwang *et al.* 2002; Baeg *et al.* 2005; Muller *et al.* 2005; Minakhina and Steward 2006; Shi *et al.* 2006; Shravage *et al.* 2013). Included in this list are Dfs uncovering *plume*, *HEM-protein*, *hairy*, *polo*, and *Autophagy-related 6* (*Atg6*) (Table S2; Shi *et al.* 2006; Shravage *et al.* 2013). The limited overlap of our screen with other known factors suggests that we may discover new modifiers of JAK/STAT signaling or of blood cell development.

### Mutation of the Hpo component Ex enhances hop^Tum-l^ melanotic tumors

**Figure 3:**
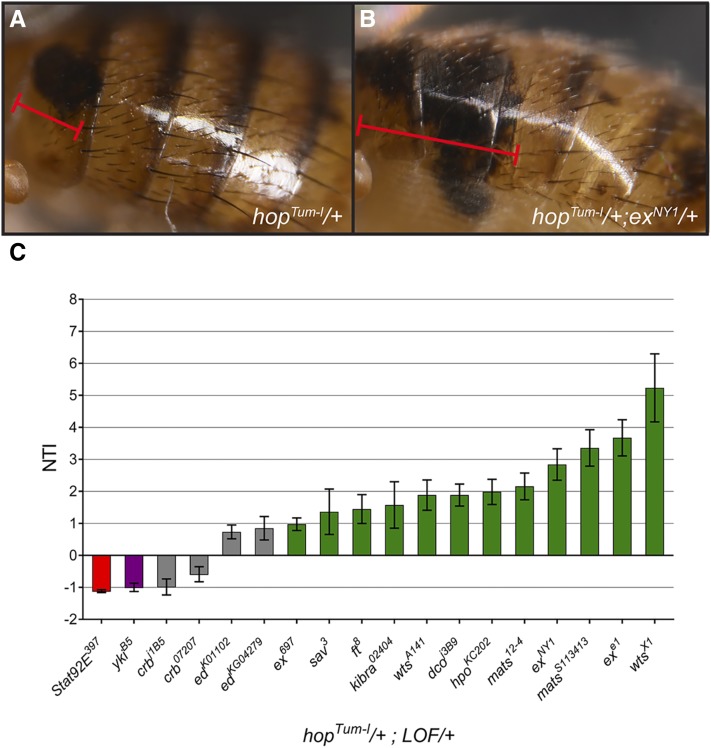
Mutations in Hpo pathway components significantly modify *hop^Tum-l^* melanotic tumors. (A and B) Light micrographs of a representative *hop^Tum-l^*/+; +/+ adult female (A) showing a typical abdominal melanotic tumor and of a representative *hop^Tum-l^*/+; *ex^NY1^*/+ adult female (B) showing enhanced tumor size. Red bracketed lines indicate the extent of the melanotic tumors in (A and B). (C) Graph of NTI ± SE for each genotype. Heterozygosity for *mats*, *ex*, *wts*, *hpo*, *kibra*, *ft*, and *dco* (green bars) enhanced tumors in *hop^Tum-l^*/+ flies, while heterozygosity for *yki* (purple bar) strongly suppresses them. Heterozygosity for *crb* and *ed* (gray bars) had no appreciable effect on tumor formation in *hop^Tum-l^*/+ flies. The red bar indicates the positive control for suppression, heterozygosity for strong hypomorphic allele *Stat92E^397^*. Hpo, Hippo; LFO, loss-of-function; NTI, normalized tumor index.

One of the strongest enhancers identified from our screen was *Df*(*2L*)*al*, which deletes the cytological region 21C1–21C7 (Figure 2). Among the genes uncovered by this deficiency, we thought that *ex* was the strongest candidate for being the underlying modifier. *ex* encodes a FERM domain-containing protein that promotes Hpo activity. Loss of *ex* leads to decreased Hpo activity and increased Yki activity (Hamaratoglu *et al.* 2006). Other laboratories have described a positive role for Yki in promoting plasmatocyte proliferation and crystal cell differentiation in the larval lymph gland (Ferguson and Martinez-Agosto 2014; Milton *et al.* 2014), supporting our hypothesis that Yki activation will promote JAK/STAT-induced tumorigenesis. We tested three different alleles of *ex* and found that two (*ex^e1^* and *ex^NY1^*) significantly enhanced the *hop^Tum-l^* tumor phenotype to similar levels seen with *Df*(*2L*)*al* (Figure 3, A–C). A third allele (*ex^697^*, an enhancer trap) mildly enhanced the *hop^Tum-l^* tumor burden (Figure 3C).

### Mutations in several Hpo pathway genes modify the tumor index in hop^Tum-l^ animals

As Ex primarily functions through the Hpo signaling pathway to regulate cell proliferation and apoptosis (Hamaratoglu *et al.* 2006), we examined our Df screen data to determine if other Dfs uncovering Hpo components were putative modifiers of *hop^Tum-l^*. We found that a Df uncovering *wts* was the strongest enhancer in the screen: *Df*(*3R*)*ED6346* (Figure 2). While this does not prove that the Hpo pathway is a general modifier of *hop^Tum-l^*, it suggests that Ex is not acting independently in this role. We sought to clarify this by testing if single hypomorphic mutations of other Hpo components were able to alter JAK/STAT-induced tumorigenesis. To accomplish this, we crossed *hop^Tum-l^* females to males carrying known Hpo pathway mutant alleles and scored the NTI in the F1 double heterozygote progeny (*i.e.*, *hop^Tum-l^*/+; mutation/+). We found that heterozygosity for alleles in multiple negative regulators of Yki activity (*wts*, *ex*, *mats*, *hpo*, *dco*, *kibra*, *sav*, and *ft*) significantly enhanced the NTI in *hop^Tum-l^* animals (Figure 3C, green bars). Moreover, heterozygosity for *yki* itself significantly suppressed the NTI in *hop^Tum-l^* animals, similar to that observed with *Stat92E* heterozygosity (Figure 3C, purple bar for *yki* and red bar for *Stat92E*). These data suggest that enhancing Yki activity in *hop^Tum-l^* animals increases the melanotic tumor burden.

### The Yki targets ban and Myc are upregulated in hop^Tum-l^ hemocytes

**Figure 4:**
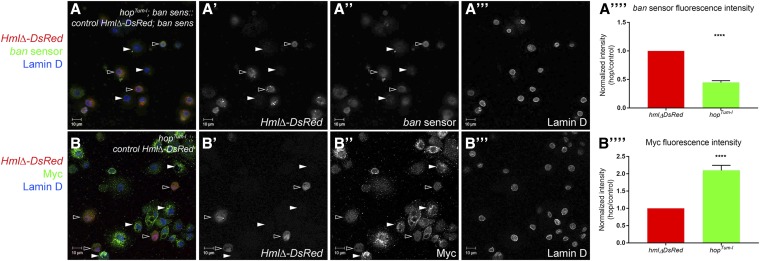
Expression of Yki targets *ban* and Myc are significantly increased in *hop^Tum-l^* hemocytes. (A and B) Mixed-cell experiments of hemolymph bleeds where control hemocytes are DsRed-positive and *hop^Tum-l^* hemocytes are DsRed-negative. Both genotypes express the *ban* sensor. Open arrowheads (A–A”) indicate DsRed-positive control hemocytes that have appreciable levels of GFP, indicating that *ban* miRNA is low in these cells. By contrast, solid arrowheads (A–A”) indicate DsRed-negative *hop^Tum-l^* hemocytes that have low levels of GFP, indicating that *ban* miRNA is higher in these cells. (A””) Quantification of fluorescence intensity of the *ban* sensor. *hop^Tum-l^* hemocytes (green bar) have significantly lower *ban* sensor levels than the control (red bar) (*P* < 0.0001). (B) Mixed-cell experiment with anti-Myc (green). Open arrowheads (B–B”) indicate DsRed-positive control hemocytes that have appreciable levels of Myc. Solid arrowheads (B–B”) indicate DsRed-negative *hop^Tum-l^* hemocytes that have higher levels of Myc than controls. (B””) Quantification of fluorescence intensity of Myc. *hop^Tum-l^* hemocytes (green bar) have significantly higher levels of Myc than the control (red bar) (*P* < 0.0001). DsRed is red and Lamin D, which marks the nucleus, is blue in (A and B). Bar, 10 μM. GFP, green fluorescent protein; miRNA, micro RNA; Yki, Yorkie.

Yki has been shown to upregulate multiple transcriptional targets, including *ban* miRNA, *Diap1*, *ex*, and *Myc* (Huang *et al.* 2005; Hamaratoglu *et al.* 2006; Thompson and Cohen 2006; Neto-Silva *et al.* 2010). In order to determine if *hop^Tum-l^* might function through any of these well-characterized Yki targets, we compared their expression on the same slide using mixed-cell experiments where we bled onto the same slide DsRed-positive control hemocytes and DsRed-negative *hop^Tum-l^* hemocytes. To monitor expression of the *ban* miRNA, we used a *ban* sensor where two copies of a 31 bp *ban* target sequence have been inserted into the 3′UTR of *Tub-eGFP* (Brennecke *et al.* 2003). When *ban* is present, GFP expression is lower. When *ban* is reduced or absent, GFP expression is higher. We crossed the *ban* sensor into both *Hml*Δ*DsRed* controls and DsRed-negative *hop^Tum-l^* animals. In mixed-cell experiments, we found that while there was variable but detectable expression of the *ban* sensor in DsRed-positive control hemocytes (Figure 4, A–A”, open arrowheads), the sensor was expressed at significantly lower levels in DsRed-negative *hop^Tum-l^* blood cells (Figure 4, A–A”, solid arrowheads, quantified in Figure 4A””, *P* < 0.0001). These data indicate that *ban* miRNA is expressed at higher levels in *hop^Tum-l^* hemocytes than controls, suggesting that Yki activity is higher in the former. We also observed significantly higher Myc protein expression in *hop^Tum-l^* hemocytes compared to controls [compare solid (*hop^Tum-l^*) arrowheads to open (control) arrowheads in Figure 4, B–B”, quantified in Figure 4B””, *P* < 0.0001]. These data also suggest that Yki activity is higher in hemocytes with sustained JAK/STAT activity. However, we observed no significant change in *Diap1-lacZ* expression [Figure S1A”, compare solid (*hop^Tum-l^*) arrowheads to open (control)], indicating that not all Yki targets are upregulated in these cells. Finally, we sought to determine whether Sd, the Yki binding partner involved in crystal cell specification in the lymph gland (Ferguson and Martinez-Agosto 2014; Milton *et al.* 2014), was expressed in peripheral blood cells and whether it had altered expression in *hop^Tum-l^* hemocytes. Using the mixed-cell experimental approach, we observed no alteration in Sd expression in DsRed-negative *hop^Tum-l^* *vs.* DsRed-positive control hemocytes [Figure S1B”, compare solid (*hop^Tum-l^*) arrowheads to open (control)]. Altogether, these data reveal that a subset of Yki target genes are upregulated in *hop^Tum-l^* hemocytes and may contribute to the tumor enhancement observed in *hop^Tum-l^* animals heterozygous for Hpo pathway components.

### Ectopic Yki signaling does not upregulate JAK/STAT activity

We reasoned that increased Yki activity may enhance *hop^Tum-l^* melanotic tumors by elevating JAK/STAT signaling in hemocytes. To test this hypothesis, we monitored Stat92E activation in DsRed-positive control hemocytes and in DsRed-negative hemocytes mis-expressing an activated form of Yki (*UAS-Yki^S168A.V5^*) (Oh and Irvine 2008). We stained this mixed-cell experiment with an antibody to Stat92E, a well-established read-out for JAK/STAT pathway activation (Flaherty *et al.* 2010). We observed no alteration in Stat92E protein staining in *Hml* > *Yki^S168A.V5^* hemocytes compared to controls [compare solid (*hop^Tum-l^*) arrowheads to open (control) arrowheads in Figure S2A–A”, quantified in Figure S2A””, *P* < 0.6253]. These data suggest that ectopic Yki activation does not upregulate Stat92E function in hemocytes and that enhanced melanotic tumor formation upon reduced Hpo activity is not a result of augmented JAK/STAT signaling by Yki.

### Ectopic Yki activation in hemocytes increases plasmatocyte proliferation but does not induce lamellocyte differentiation

**Figure 5:**
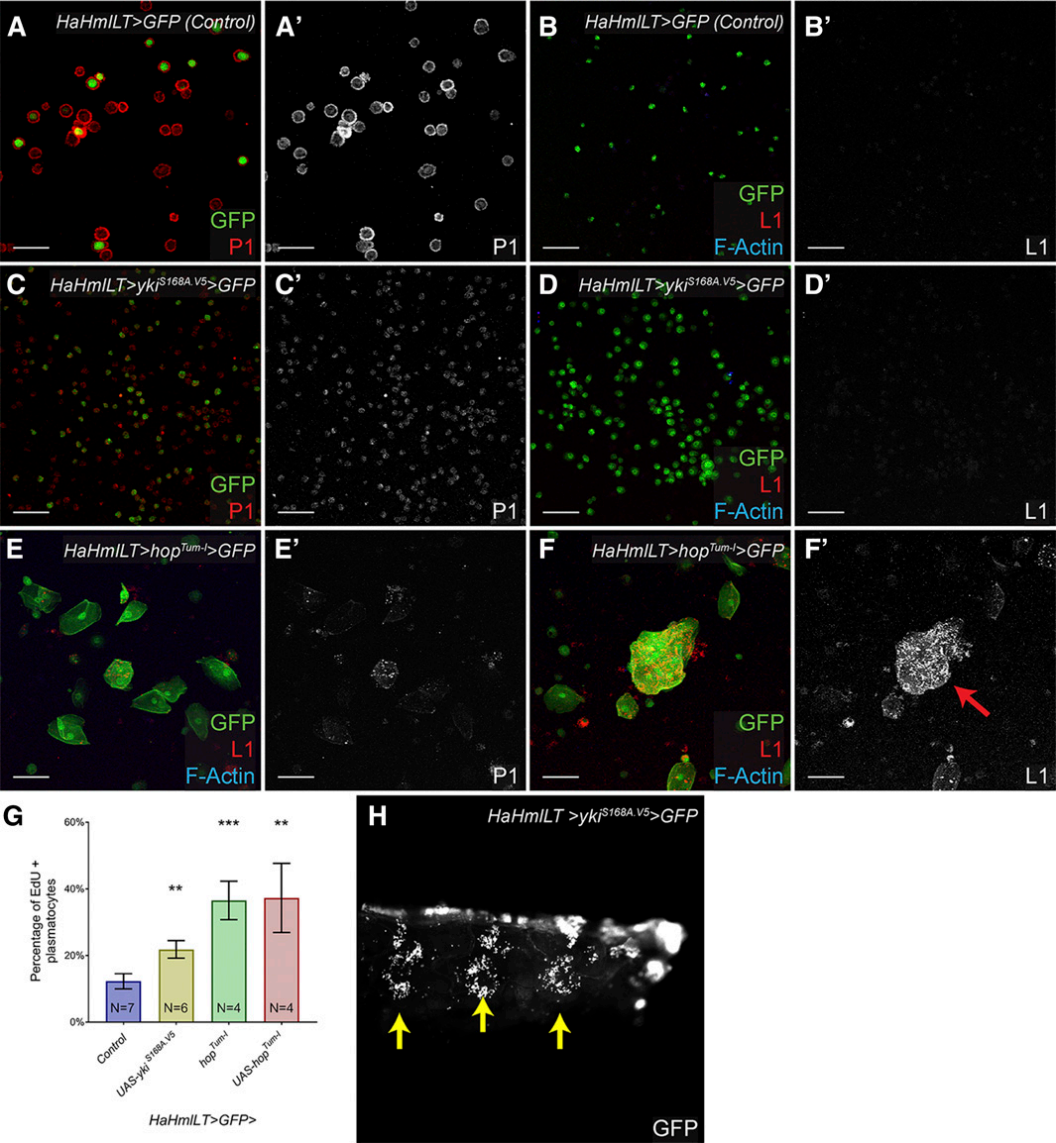
Hematopoietic activation of Yki increases plasmatocyte proliferation but does not induce lamellocyte differentiation. (A and B) Hemolymph bleeds of *HaHmlLT* > *GFP* control animals reveal GFP-positive hemocytes, most of which are P1-expressing plasmatocytes (A) and none of which are L1-expressing lamellocytes (B). (C and D) Bleeds from *HaHmlLT* > *Yki^S168A.V5^* animals appear to have more P1-positive plasmatocytes than controls (C) but no L1-positive lamellocytes (D). (E and F) Mis-expression of *UAS-hop^Tum-l^* by *HaHmlLT-Gal4* leads to robust lamellocyte differentiation (E) and the formation of microtumors (F, arrow). (G) EdU *ex vivo* labeling of circulating hemocytes from control *HaHmlLT* > *GFP* (blue bar), *HaHmlLT* > *Yki^S168A.V5^* (yellow bar), *hop^Tum-l^* (green bar), and *HaHmlLT* > *hop^Tum-l^* (red bar) larvae. There are significantly more EdU-positive, P1-positive cells in *HaHmlLT* > *Yki^S168A.V5^* (*P* < 0.01), *hop^Tum-l^* (*P* < 0.001), and *HaHmlLT* > *hop^Tum-l^* (*P* < 0.01) bleeds than in the control. (H) Residence of hemocytes in larval hematopoietic pockets in *HaHmlLT* > *Yki^S168A.V5^* animals is similar to controls (compare with Figure 1M). In (A–F), the hematopoietic lineage is green. P1 is red in (A) and (C), and L1 is red and F-actin is blue in (B), (D), (E), and (F). Bar, 50 μM. EdU, 5-ethynyl-2′-deoxyuridine; GFP, green fluorescent protein; UAS, upstream activating sequence; Yki, Yorkie.

One possibility is that sustained Yki activation in peripheral blood cells induces plasmatocyte proliferation and lamellocyte differentiation. This hypothesis is consistent with the known role of Yki in promoting proliferation of plasmatocytes in the lymph gland (Ferguson and Martinez-Agosto 2014; Milton *et al.* 2014). However, Yki is not required for lamellocyte differentiation in response to wasp infestation, suggesting that Yki activation is not sufficient to induce the lamellocyte fate (Ferguson and Martinez-Agosto 2014). To test our hypothesis directly, we mis-expressed activated Yki (*UAS-Yki^S168A.V5^*) using the *HaHmlLT-Gal4* driver. Hemolymph from larvae with hematopoietic Yki activation contained numerous plasmatocytes compared to controls, but we observed no lamellocytes in these larvae (Figure 5, C–D). Additionally, these animals (*HaHmlLT* > *Yki^S168A.V5^*) did not display melanotic tumors. By contrast, hematopoietic mis-expression of *UAS-hop^Tum-l^* with *HaHmlLT-Gal4* induced robust lamellocyte differentiation and the formation of microtumors (Figure 5, E and F, arrow). These results demonstrate that Yki activation in hematopoietic tissue is not sufficient to induce lamellocyte differentiation. Furthermore, they suggest that Yki activity expands the circulating plasmatocyte pool by increasing proliferation. To test this hypothesis, we *ex vivo* labeled circulating plasmatocytes with the S phase marker EdU in control, *HaHmlLT* > *Yki^S168A.V5^*, *hop^Tum-l^*, and *HaHmlLT* > *hop^Tum-l^* larvae. There were significantly more EdU-positive, P1-positive cells in *HaHmlLT* > *Yki^S168A.V5^* larvae compared to control [Figure 5G, blue bar (Control), yellow bar (*UAS-Yki^S168A.V5^*), *P* < 0.01]. We also observed significantly more cycling plasmatocytes in *hop^Tum-l^* and *HaHmlLT* > *hop^Tum-l^* larvae compared to control [Figure 5G, green bar (*hop^Tum-l^*), pink red (*UAS-hop^Tum-l^*) *P* < 0.001 for *hop^Tum-l^* and *P* < 0.01 for *UAS-hop^Tum-l^*]. Furthermore, unlike *hop^Tum-l^* and *HaHmlLT* > *hop^Tum-l^* larvae, which had altered hemocyte residence in hematopoietic pockets (Figure 1, N and O), *HaHmlLT* > *Yki^S168A.V5^* larvae had hematopoietic pockets that appeared similar to control larvae (compare Figure 5H to Figure 1M). These results indicate that ectopic Yki activation was not sufficient to perturb hemocyte residency in pockets. Taken together, these results indicate that Yki activation in hemocytes increases the rate of plasmatocyte proliferation but does not directly promote lamellocyte differentiation.

### Hematopoietic depletion of Yki reduces the tumor burden in hop^Tum-l^ animals

**Figure 6:**
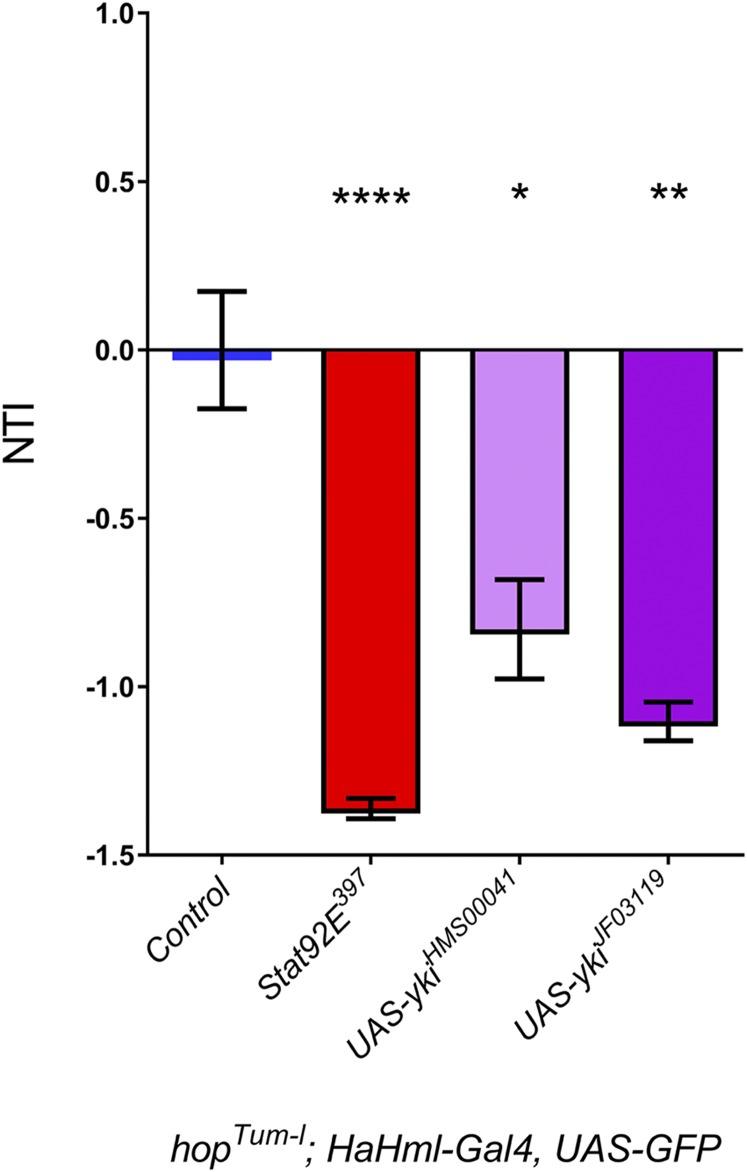
Hematopoietic depletion of Yki significantly lowers the tumor burden in *hop^Tum-l^* animals. The first bar (blue bar) indicates the NTI in *hop^Tum-l^*/+; *HaHml* > *GFP/+* adult females (labeled “control”). Heterozygosity for *Stat92E* (red bar, genotype: *hop^Tum-l^*/+; *HaHml* > *GFP/+*; *Stat92E^397^*/+) strongly suppresses the NTI (*P* < 0.0001). Hematopoietic depletion of Yki by RNAi (light purple bar, genotype: *hop^Tum-l^*/+; *HaHml* > *GFP/UAS-yki^HMS00041^*; dark purple bar *hop^Tum-l^*/+; *HaHml* > *GFP/UAS-yki^JF03119^*) significantly reduces the NTI (*P* < 0.0286 for *yki^HMS00041^* and *P* < 0.0023 for *yki^JF03119^*). GFP, green fluorescent protein; NTI, normalized tumor index; UAS, upstream activating sequence; Yki, Yorkie.

Finally, we wanted to determine the hematopoietic importance of Yki in JAK/STAT-dependent melanotic tumors. To accomplish this, we depleted Yki from *hop^Tum-l^* animals by RNAi using *HaHml-Gal4* that targets the entire hematopoietic lineage. Two independent UAS-RNAi lines targeting Yki (*HMS00041* and *JF03119*) significantly reduced the NTI of *hop^Tum-l^* adult animals (Figure 6, light and dark purple bars; *P* < 0.0283 for *HMS00041* and *P* < 0.0023 for *JF03119*). However, neither suppressed the NTI to the extent observed with heterozygosity for a hypomorphic *Stat92E* mutation (Figure 6, red bar; *P* < 0.0001). While this could be due to inefficiency of RNAi, we favor the interpretation that knockdown of Yki suppresses the enhanced proliferation of hemocytes in *hop^Tum-l^* animals, while reduction of *Stat92E* in this background suppresses plasmatocyte proliferation and lamellocyte differentiation.

## Discussion

Using an F1 genetic modifier screen, we have identified Dfs uncovering *ex* and *wts* as the strongest enhancers of JAK/STAT-induced melanotic tumors. Furthermore, we determined that reducing the genetic dose of *ex*, *wts*, or other negative regulators of Yki significantly enhanced the tumor burden in *hop^Tum-l^* animals, while reducing *yki* significantly suppressed it. We showed that the Yki targets *ban* and Myc are upregulated in *hop^Tum-l^* hemocytes compared to controls and that knockdown of Yki in the hemocyte lineage suppresses the *hop^Tum-l^* phenotype. Future work will need to determine how Yki activity is upregulated in *hop^Tum-l^* hemocytes. One possibility is that ectopic JAK/STAT signaling inhibits the expression or function of a negative regulator of Yki (*e.g.*, Ex, Hpo, or Wts). Another potential mechanism is that JAK/STAT signaling promotes the expression or function of a positive regulator of Yki, possibly even Yki itself. One issue that arises from our study is how Hpo signaling might act in circulating hemocytes, as the pathway has generally been studied in the context of cell-cell contact in epithelial tissue (Irvine and Harvey 2015). Notably, Yki has been reported to regulate Myc through *ban* miRNA in glial cell proliferation (Reddy and Irvine 2011), suggesting that Hpo signaling plays a role in nonepithelial cells. Moreover, this regulation appears to be through Merlin–Hpo, not through other upstream components like Ex, suggesting that Hpo–Yki signaling may be regulated differently in certain cell types or tissues.

We plan to map, at the level of the gene, other modifiers identified through this screen in the future. While we identified interactors common to other JAK/STAT gain-of-function screens, numerous hits were unique to ours. The differences between this report and a previous screen for JAK/STAT-mediated tumorigenesis might be due to (1) the parameters we used for scoring and defining significance, resulting in differences in classification, and (2) the use of earlier Df kits by Shi *et al.* (2006) that contain Dfs with different coverage, thus similar regions may not be identified. Going forward, we will also need to consider interacting Dfs/underlying modifiers in light of our new finding that residence in hematopoietic pockets is perturbed in *hop^Tum-l^* larvae and results from autonomous JAK/STAT activation in the hematopoietic compartment. Subsequent work will be needed to determine whether homing to pockets, length of residence in the pockets, cell-cell contact in pockets, and/or transit between pockets and circulation are altered in *hop^Tum-l^* animals, and whether modifiers identified by the screen impact any of these parameters.

Reports from other laboratories have shown that dysregulation of Jun N-terminal Kinase (JNK), Toll (Tl), and Ras causes lamellocyte differentiation and melanotic tumors (Lemaitre *et al.* 1995; Luo *et al.* 1995; Qiu *et al.* 1998; Asha *et al.* 2003; Zettervall *et al.* 2004; Minakhina and Steward 2006). In the future, it will be of interest to determine if Hpo signaling specifically plays a role in *hop^Tum-l^*-induced melanotic tumors, or if Hpo has a broader role in melanotic tumors induced by other pathways. Finally, it would be important to determine if increased YAP activity is observed in samples from MPN patients or from mice harboring the *JAK2^V617F^* mutation.

## Supplementary Material

Supplemental material is available online at www.g3journal.org/lookup/suppl/doi:10.1534/g3.117.044172/-/DC1.
