# Supplementary figures and images for: A Genetic Screen Reveals an Unexpected Role for Yorkie Signaling in JAK/STAT-Dependent Hematopoietic Malignancies in Drosophila melanogaster
Source: G3 (Bethesda). 2017 Jun 15;7(8):2427–38. doi: 10.1534/g3.117.044172 (PMC5555452; doi:10.1534/g3.117.044172)

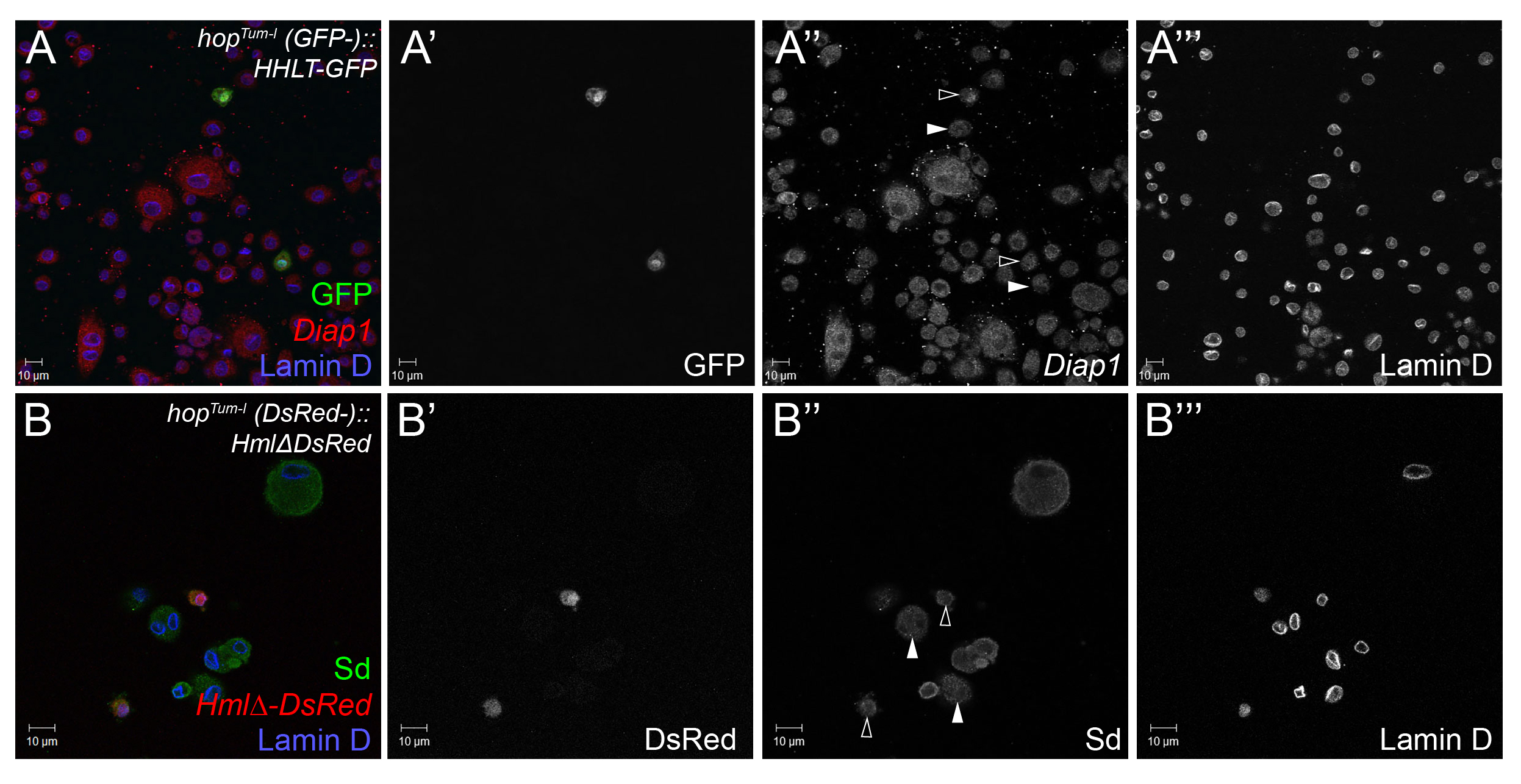

Supplement: Supplementary file 1 [file 2427FigureS1.jpg]

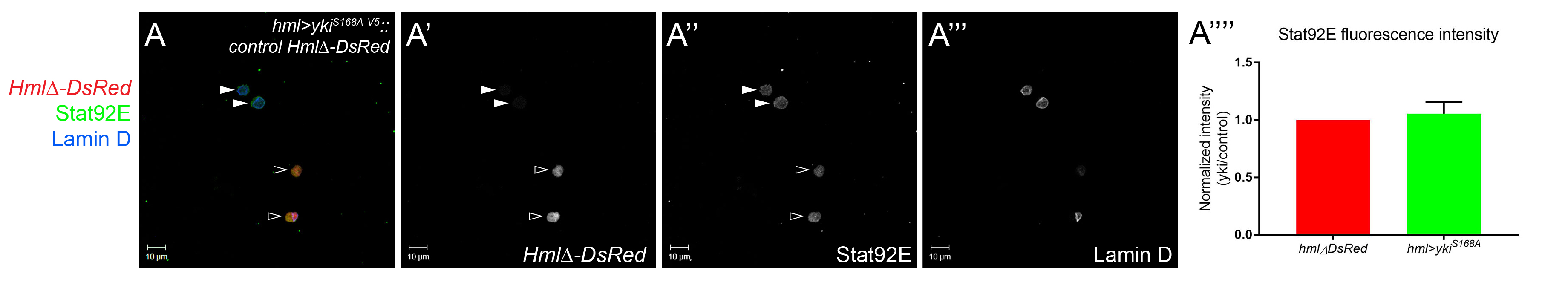

Supplement: Supplementary file 2 [file 2427FigureS2.jpg]
