# Supplementary material for: A Genetic Screen Reveals an Unexpected Role for Yorkie Signaling in JAK/STAT-Dependent Hematopoietic Malignancies in Drosophila melanogaster
Source: G3 (Bethesda). 2017 Jun 15;7(8):2427–38. doi: 10.1534/g3.117.044172 (PMC5555452; doi:10.1534/g3.117.044172)
Supplement: Supplementary file 3 [file 2427FileS1.docx]

**Figure S1. Yki target *Diap1* and Yki interacting protein Sd are not altered in *hop^Tum-l^* hemocytes.** (A) A mixed-cell experiment of larval hemolymph where control hemocytes are GFP-positive and *hop^Tum-l^* hemocytes are GFP-negative. (A) Both genotypes express the enhancer trap *Diap1-lacZ*. Open and solid arrowheads (A”) show that control and *hop^Tum-l^* hemocytes, respectively, have similar levels of Diap1 (β-Gal, red). (B) A mixed-cell experiment where control hemocytes are DsRed-positive and *hop^Tum-l^* hemocytes are DsRed-negative. The cells were stained with anti-Sd (green). Open and solid arrowheads (B”) show that control and *hop^Tum-l^* hemocytes, respectively, have similar levels of Sd. GFP is green in A, DsRed is red in B and Lamin D is blue in A, B. Scale bars indicate 10 μM.
