# Supplementary material for: A Genetic Screen Reveals an Unexpected Role for Yorkie Signaling in JAK/STAT-Dependent Hematopoietic Malignancies in Drosophila melanogaster
Source: G3 (Bethesda). 2017 Jun 15;7(8):2427–38. doi: 10.1534/g3.117.044172 (PMC5555452; doi:10.1534/g3.117.044172)
Supplement: Supplementary file 4 [file 2427FileS2.docx]

**Figure S2. Ectopic Yki signaling does not upregulate Stat92E activity.** (A) A mixed-cell experiment of larval hemolymph where control hemocytes are DsRed-positive and *Hml>Yki^S168A-V5^* hemocytes are DsRed-negative. (A) Both genotypes were stained with an antibody specific for Stat92E (green). Open and solid arrowheads (A”) show that control and *Hml>Yki^S168A-V5^* hemocytes, respectively, have similar levels of Stat92E. (B) Quantification of fluorescence intensity of Stat92E. There is no significant difference between the intensity of Stat92E in control (red bar) and *Hml>Yki^S168A-V5^* hemocytes (green bar) (P<0.6253). In A, DsRed is red and Lamin D is blue. Scale bars indicate 10 μM.
