# Supplementary material for: A Genetic Screen Reveals an Unexpected Role for Yorkie Signaling in JAK/STAT-Dependent Hematopoietic Malignancies in Drosophila melanogaster
Source: G3 (Bethesda). 2017 Jun 15;7(8):2427–38. doi: 10.1534/g3.117.044172 (PMC5555452; doi:10.1534/g3.117.044172)
Supplement: Supplementary file 5 [file 2427TableS1.docx]

| **Table S1. Full dataset for *hop^Tum-l^* Df screen.** Data are presented as normalized TI for chromosome arms (2L, 2R, 3L, 3R) arranged according to cytology. | | | |
| --- | --- | --- | --- |
| *hop^Tum-l^* 2L Deficiency Screen Results | | | |
| **Df symbol** | **Cytology** | **Interaction** | **Normalized TI** |
| Df(2L)ED50001 | 21A1;21B1 | NE | -0.080 |
| Df(2L)ED5878 | 21B1;21B3 | NE | 0.441 |
| Df(2L)ED19 | 21B3;21B7 | NE | 0.514 |
| Df(2L)BSC454 | 21B7;21B8 | NE | -0.059 |
| Df(2L)BSC106 | 21B7;21C2 | NE | 0.339 |
| Df(2L)al | 21C1--21C7 | En | 4.406 |
| Df(2L)BSC107 | 21C2;21E2 | NE | 0.614 |
| Df(2L)ast2 | 21D1--2;22B2--3 | NE | -0.468 |
| Df(2L)ED105 | 21E2;22A1 | NE | 0.343 |
| Df(2L)ED94 | 21E2;21E3 | NE | -0.115 |
| Df(2L)Exel6005 | 22A3;22B1 | NE | -0.699 |
| Df(2L)BSC688 | 22B1;22D6 | NE | 0.813 |
| Df(2L)BSC455 | 22D5;22E1 | NE | -0.582 |
| Df(2L)Exel7011 | 22E1;22F3 | Lethal |  |
| Df(2L)C144 | 22F4;23C3 | En | 1.612 |
| Df(2L)ED136 | 22F4;23A3 | NE | -0.271 |
| Df(2L)Exel6277 | 23A2;23B1 | NE | 0.286 |
| Df(2L)BSC692 | 23B3;23B7 | NE | -0.526 |
| Df(2L)BSC180 | 23B7;23C3 | Su | -1.205 |
| Df(2L)ED4651 | 23B8;23F3 | NE | 0.000 |
| Df(2L)drm-P2 | 23F3--4;24A1--2 | NE | -0.636 |
| Df(2L)BSC292 | 23F6;24A2 | NE | -0.469 |
| Df(2L)ED247 | 24A2;24C3 | NE | 0.491 |
| Df(2L)ed1 | 24A2;24D4 | NE | -0.442 |
| Df(2L)Exel6009 | 24C3;24C8 | NE | 0.406 |
| Df(2L)BSC165 | 24D4;24D8 | NE | -0.463 |
| Df(2L)BSC295 | 24D4;24F3 | Su | -1.116 |
| Df(2L)M24F-B | 24E1--2;24F6--7 | NE | -0.813 |
| Df(2L)ED250 | 24F4;25A7 | NE | 0.042 |
| Df(2L)ED7853 | 25A3;25B10 | NE | -0.165 |
| Df(2L)tkv3 | 25A4--5;25D5 | NE | -0.982 |
| Df(2L)BSC172 | 25B10;25C1 | NE | 0.350 |
| Df(2L)BSC110 | 25C1;25C4 | NE | -0.012 |
| Df(2L)BSC109 | 25C4;25C8 | NE | 0.779 |
| Df(2L)Exel6011 | 25C8;25D5 | NE | 0.426 |
| Df(2L)Exel6012 | 25D5;25E6 | En | 2.201 |
| Df(2L)BSC169 | 25E5;25F3 | NE | 0.261 |
| Df(2L)ED334 | 25F2;26B2 | NE | 0.349 |
| Df(2L)ED385 | 26B1;26D7 | NE | -0.251 |
| Df(2L)BSC6 | 26D3--E1;26F4--7 | En | 1.626 |
| Df(2L)BSC354 | 26D7;26E3 | NE | 0.097 |
| Df(2L)BSC188 | 26F1;27A2 | Su | -1.498 |
| Df(2L)BSC291 | 27D6;27F2 | NE | -0.366 |
| Df(2L)BSC233 | 27F3;28D2 | NE | -0.215 |
| Df(2L)BSC142 | 28C3;28D3 | NE | -0.501 |
| Df(2L)Exel7034 | 28E1;28F1 | NE | -0.354 |
| Df(2L)BSC227 | 28E8;29B1 | NE | 0.141 |
| Df(2L)BSC111 | 28F5;29B1 | NE | 1.741 |
| Df(2L)ED629 | 29B4;29E4 | En | 2.065 |
| Df(2L)BSC204 | 29D5;29F8 | NE | -0.400 |
| Df(2L)ED678 | 29F5;30B12 | NE | -0.153 |
| Df(2L)ED690 | 30B3;30E4 | En | 2.618 |
| Df(2L)BSC17 | 30C3--5;30F1 | En | 3.189 |
| Df(2L)BSC240 | 30C7;30F2 | NE | -0.393 |
| Df(2L)BSC50 | 30F4--5;31B1--4 | NE | 3.117 |
| Df(2L)BSC689 | 30F5;31B1 | NE | -0.647 |
| Df(2L)BSC143 | 31B1;31D9 | En | 2.099 |
| Df(2L)J39 | 31C--D;32D--E | Lethal |  |
| Df(2L)BSC208 | 31D7;31D11 | NE | 0.201 |
| Df(2L)BSC209 | 31D7;31E1 | NE | -0.976 |
| Df(2L)ED8142 | 31E1;32A4 | NE | 0.075 |
| Df(2L)BSC214 | 31F5;32B4 | NE | 0.724 |
| Df(2L)BSC213 | 32B1;32C1 | NE | -0.248 |
| Df(2L)BSC145 | 32C1;32C1 | NE | 0.414 |
| Df(2L)BSC241 | 32C1;32F2 | NE | 0.672 |
| Df(2L)BSC244 | 32F2;33B6 | NE | -0.920 |
| Df(2L)ED761 | 33A2;33E5 | NE | 0.514 |
| Df(2L)ED775 | 33B8;34A3 | En | 1.314 |
| Df(2L)BSC277 | 34A1;34B2 | Su | -1.101 |
| Df(2L)BSC892 | 34A5;34B9 | NE | -0.424 |
| Df(2L)BSC159 | 34B4;34C4 | Su | -1.049 |
| Df(2L)BSC812 | 34B11;34E1 | NE | -0.832 |
| Df(2L)BSC252 | 34D1;34F1 | En | 1.538 |
| Df(2L)ED793 | 34E4;35B4 | NE | 0.223 |
| Df(2L)ED3 | 35B2;35D1 | NE | 0.635 |
| Df(2L)ED1050 | 35B8;35D4 | NE | 0.402 |
| Df(2L)r10 | 35D1;36A6--7 | NE | -0.006 |
| Df(2L)BSC690 | 35D4;35D4 | NE | -0.279 |
| Df(2L)Exel6038 | 35D6;35E2 | NE | -0.463 |
| Df(2L)BSC278 | 35E1;35F1 | NE | 0.184 |
| Df(2L)BSC781 | 35F1;36A1 | NE | -0.230 |
| Df(2L)ED1102 | 35F12;36A10 | NE | 0.518 |
| Df(2L)H20 | 36A8--9;36E1--2 | NE | 0.685 |
| Df(2L)ED1161 | 36A10;36C9 | NE | 0.236 |
| Df(2L)BSC148 | 36C8;36E3 | NE | 0.390 |
| Df(2L)Exel7070 | 36E2;36E6 | NE | 0.067 |
| Df(2L)BSC256 | 36E3;36F2 | NE | -0.689 |
| Df(2L)Exel8038 | 36E5;36F5 | NE | -0.317 |
| Df(2L)BSC149 | 36F5;36F10 | Su | -1.279 |
| Df(2L)ED1203 | 36F7;37C5 | En | 2.909 |
| Df(2L)ED1272 | 37C5;38A2 | NE | 0.183 |
| Df(2L)ED1303 | 37E5;38C6 | En | 1.951 |
| Df(2L)ED1315 | 38B4;38F5 | NE | -0.364 |
| Df(2L)ED1378 | 38F1;39D2 | En | 1.331 |
| Df(2L)ED1473 | 39B4;40A5 | NE | 0.076 |
|  |  |  |  |
|  |  |  |  |
|  |  |  |  |
|  |  |  |  |
|  |  |  |  |
|  |  |  |  |
|  |  |  |  |
|  |  |  |  |
|  |  |  |  |

| *hop^Tum-l^* 2R Deficiency Screen Results | | | |
| --- | --- | --- | --- |
| **Df symbol** | **Cytology** | **Interaction** | **Normalized TI** |
| Df(2R)M40A10 | h38R;h46 (Df) | En | 2.013 |
| Df(2R)BSC630 | 41D3;41F11 (Df) | NE | -0.858 |
| Df(2R)BSC889 | 41F11;42A13 (Df) | NE | -0.589 |
| Df(2R)ED1484 | 42A2;42A14 (Df) | NE | 0.600 |
| Df(2R)ED1612 | 42A13;42E6 (Df) | NE | 0.498 |
| Df(2R)ED1673 | 42E1;43D3 (Df) | NE | -0.255 |
| Df(2R)ED1715 | 43A4;43F1 (Df) | NE | 0.453 |
| Df(2R)ED1725 | 43E4;44B5 (Df) | En | 1.268 |
| Df(2R)BSC267 | 44A4;44C4 (Df) | NE | 0.261 |
| Df(2R)ED1742 | 44B8;44E3 (Df) | NE | 0.307 |
| Df(2R)ED1770 | 44D5;45B4 (Df) | NE | -0.744 |
| Df(2R)ED1791 | 44F7;45F1 (Df) | NE | 0.581 |
| Df(2R)BSC280 | 45C4;45F4 (Df) | NE | -0.625 |
| Df(2R)BSC132 | 45F6;46B4 (Df) | NE | -0.324 |
| Df(2R)BSC298 | 46B2;46C7 (Df) | NE | 0.176 |
| Df(2R)X1Mef2X1 | 46C2;47A1 (Df) | NE | -0.346 |
| Df(2R)BSC152 | 46C1;46D6 (Df) | NE | -0.333 |
| Df(2R)BSC303 | 46E1;46F3 (Df) | NE | -0.276 |
| Df(2R)BSC281 | 46F1;47A9 (Df) | NE | -0.320 |
| Df(2R)BSC595 | 47A3;47F1 (Df) | NE | 0.248 |
| Df(2R)ED2219 | 47D6;48B6 (Df) | En | 3.017 |
| Df(2R)ED2247 | 48A3;48D5 (Df) | NE | -0.360 |
| Df(2R)BSC199 | 48C5;48E4 (Df) | NE | -0.346 |
| Df(2R)BSC699 | 48D7;48E6 (Df) | NE | 0.183 |
| Df(2R)BSC495 | 48F1;49A1 (Df) | NE | -0.458 |
| Df(2R)Exel6061 | 48F1;49A6 (Df) | NE | -0.392 |
| Df(2R)BSC305 | 49A4;49A10 (Df) | NE | -0.397 |
| Df(2R)BSC880 | 49A9;49E1 (Df) | NE | -0.523 |
| Df(2R)BSC485 | 49B10;49E6 (Df) | NE | 0.407 |
| Df(2R)CX1 | 49C1;50D3-50D5 (Df) | NE | 0.696 |
| Df(2R)Exel6062 | 49E6;49F1 (Df) | NE | -0.318 |
| Df(2R)BSC273 | 49F4;50A13 (Df) | NE | -0.189 |
| Df(2R)Exel8057 | 49F1;49F10 (Df) | NE | 0.230 |
| Df(2R)BSC274 | 50A7;50B4 (Df) | NE | 0.010 |
| Df(2R)BSC307 | 50B6;50C18 (Df) | NE | 0.430 |
| Df(2R)BSC361 | 50C3;50F1 (Df) | NE | -0.406 |
| Df(2R)BSC383 | 50C6;50D2 (Df) | NE | 0.210 |
| Df(2R)Exel7130 | 50D4;50E4 (Df) | NE | -0.063 |
| Df(2R)Exel7131 | 50E4;50F6 (Df) | En | 2.245 |
| Df(2R)ED2354 | 50E6;51B1 (Df) | En | 1.451 |
| Df(2R)Exel6284 | 51B1;51C2 (Df) | NE | -0.192 |
| Df(2R)BSC429 | 51C2;51D1 (Df) | NE | -0.130 |
| Df(2R)BSC651 | 51C5;51E2 (Df) | NE | 0.027 |
| Df(2R)ED2426 | 51E2;52B1 (Df) | NE | -0.194 |
| Df(2R)BSC427 | 52A10;52D2 (Df) | NE | -0.746 |
| Df(2R)BSC308 | 52B5;52D15 (Df) | NE | 0.579 |
| Df(2R)ED2457 | 52D11;52E7 (Df) | NE | 0.816 |
| Df(2R)ED2487 | 52E6;53C4 (Df) | En | 1.092 |
| Df(2R)BSC550 | 53C1;53C6 (Df) | NE | -0.305 |
| Df(2R)Exel7144 | 53C8;53D2 (Df) | NE | -0.564 |
| Df(2R)Exel6064 | 53C11;53D11 (Df) | NE | -0.192 |
| Df(2R)ED2747 | 53D11;53F8 (Df) | NE | 0.330 |
| Df(2R)BSC331 | 53D14;54A1 (Df) | NE | -0.028 |
| Df(2R)Exel6066 | 53F8;54B6 (Df) | NE | 0.965 |
| Df(2R)BSC161 | 54B2;54B17 (Df) | NE | -0.064 |
| Df(2R)BSC335 | 54B16;54C3 (Df) | NE | -0.183 |
| Df(2R)ED3385 | 54C3;54D4 (Df) | NE | -0.365 |
| Df(2R)Exel7149 | 54C10;54D5 (Df) | NE | -0.090 |
| Df(2R)BSC347 | 54D2;54E9 (Df) | NE | 0.083 |
| Df(2R)14H10W35 | 54E5-54E7;55B5-55B7 (Df) | NE | 0.230 |
| Df(2R)ED3610 | 54F1;55C8 (Df) | NE | 0.147 |
| Df(2R)ED3683 | 55C2;56C4 (Df) | NE | -0.316 |
| Df(2R)Exel6069 | 56B5;56C11 (Df) | NE | -0.018 |
| Df(2R)BSC135 | 56C11;56D5 (Df) | NE | -0.358 |
| Df(2R)BSC782 | 56D8;56D14 (Df) | NE | -0.148 |
| Df(2R)ED3728 | 56D10;56E2 (Df) | En | 1.115 |
| Df(2R)BSC883 | 56E1;56F11 (Df) | NE | -0.189 |
| Df(2R)Exel7162 | 56F11;56F16 (Df) | NE | -0.364 |
| Df(2R)BSC19 | 56F12-56F14;57A4 (Df) | NE | -0.389 |
| Df(2R)BSC701 | 56F15;57A9 (Df) | NE | -0.116 |
| Df(2R)BSC702 | 57A2;57B3 (Df) | NE | -0.496 |
| Df(2R)ED3791 | 57B1;57D4 (Df) | NE | -0.723 |
| Df(2R)BSC885 | 57D2;57D10 (Df) | NE | -0.471 |
| Df(2R)BSC821 | 57D10;57E6 (Df) | NE | 0.095 |
| Df(2R)BSC664 | 57D12;58A3 (Df) | NE | -0.001 |
| Df(2R)BSC597 | 58A2;58F1 (Df) | NE | -0.215 |
| Df(2R)X58-12 | 58D1-58D2;59A (Df) | NE | -0.544 |
| Df(2R)BSC598 | 58F3;59A1 (Df) | NE | -0.581 |
| Df(2R)BSC787 | 58F4;59B1 (Df) | NE | -0.031 |
| Df(2R)BSC599 | 59B1;59B3 (Df) | NE | -0.464 |
| Df(2R)BSC784 | 59B4;59B6 (Df) | NE | -0.071 |
| Df(2R)BSC769 | 59B7;59D9 (Df) | NE | -0.321 |
| Df(2R)BSC661 | 59D8;59F5 (Df) | NE | -0.357 |
| Df(2R)BSC136 | 59F5;60B6 (Df) | NE | 0.135 |
| Df(2R)BSC356 | 60B8;60C4 (Df) | NE | -0.256 |
| Df(2R)BSC780 | 60C2;60D14 (Df) | NE | -0.692 |
| Df(2R)BSC604 | 60D4;60E11 (Df) | NE | 0.104 |
| Df(2R)Kr10 | 60E10;60F5 (Df) | NE | -0.212 |
| Df(2R)BSC608 | 60E11;60F2 (Df) | NE | -0.139 |
| Df(2R)ED50004 | 60F5;2Rt (Df) | NE | 0.367 |
| Df(2R)M40A10 | h38R;h46 (Df) | En | 2.013 |

| *hop^Tum-l^* 3L Deficiency Screen Results | | | |
| --- | --- | --- | --- |
| **Df symbol** | **Cytology** | **Interaction** | **Normalized TI** |
| Df(3L)ED201 | 61B1;61C1 | NE | 0.354 |
| Df(3L)BSC362 | 61C1;61C7 | NE | -0.101 |
| Df(3L)Exel6085 | 61C3;61C9 | NE | -0.454 |
| Df(3L)ED4196 | 61C7;62A2 | En | 3.060 |
| Df(3L)BSC289 | 61F6;62A9 | NE | 0.723 |
| Df(3L)BSC800 | 62A9;62A9 | NE | -0.555 |
| Df(3L)BSC181 | 62A11;62B7 | NE | -0.407 |
| Df(3L)Aprt-32 | 62B1;62E3 | NE | 0.133 |
| Df(3L)ED4287 | 62B4;62E5 | NE | -0.417 |
| Df(3L)BSC119 | 62E7;62F5 | NE | 0.196 |
| Df(3L)BSC23 | 62E8;63B5--6 | NE | -0.874 |
| Df(3L)M21 | 62F;63D | NE | -0.449 |
| Df(3L)Exel6092 | 62F5;63A3 | NE | -0.430 |
| Df(3L)BSC671 | 63A2;63B11 | NE | -0.265 |
| Df(3L)BSC672 | 63A7;63B12 | NE | -0.603 |
| Df(3L)ED4293 | 63C1;63C1 | NE | 0.506 |
| Df(3L)ED208 | 63C1;63F5 | NE | -0.236 |
| Df(3L)BSC368 | 63F1;64A4 | NE | 0.403 |
| Df(3L)ED4341 | 63F6;64B9 | NE | -0.024 |
| Df(3L)ED210 | 64B9;64C13 | NE | -0.380 |
| Df(3L)ZN47 | 64C;65C | En | 2.927 |
| Df(3L)BSC371 | 64C1;64E1 | NE | -0.561 |
| Df(3L)BSC884 | 64D6;64E7 | NE | -0.055 |
| Df(3L)BSC410 | 64E7;65B3 | NE | -0.474 |
| Df(3L)BSC411 | 65A2;65C1 | NE | 0.123 |
| Df(3L)Exel6109 | 65C3;65D3 | NE | -0.061 |
| Df(3L)BSC27 | 65D4--5;65E4--6 | NE | 0.942 |
| Df(3L)BSC224 | 65D5;65E6 | NE | -0.193 |
| Df(3L)BSC117 | 65E9;65F5 | Su | -1.678 |
| Df(3L)BSC33 | 65E10--F1;65F2--6 | NE | 0.611 |
| Df(3L)Exel8104 | 65F7;66A4 | NE | -0.250 |
| Df(3L)BSC375 | 66A3;66A19 | NE | -0.007 |
| Df(3L)BSC388 | 66A8;66B11 | NE | -0.492 |
| Df(3L)Exel6112 | 66B5;66C8 | NE | -0.218 |
| Df(3L)BSC815 | 66C3;66D4 | NE | -0.776 |
| Df(3L)BSC389 | 66C12;66D8 | NE | -0.476 |
| Df(3L)BSC816 | 66D9;66D12 | En | 1.512 |
| Df(3L)ED4421 | 66D12;67B3 | NE | -0.301 |
| Df(3L)AC1 | 67A2;67D11--13 | NE | 1.045 |
| Df(3L)BSC113 | 67B1;67B5 | NE | 0.301 |
| Df(3L)BSC391 | 67B7;67C5 | NE | -0.404 |
| Df(3L)BSC118 | 67B11;67C5 | NE | 0.387 |
| Df(3L)BSC392 | 67C4;67D1 | NE | -0.506 |
| Df(3L)BSC673 | 67C7;67D10 | NE | -0.547 |
| Df(3L)ED4457 | 67E2;68A7 | NE | -0.293 |
| Df(3L)ED4470 | 68A6;68E1 | NE | 0.277 |
| Df(3L)ED4475 | 68C13;69B4 | NE | 0.123 |
| Df(3L)BSC730 | 68F7;69E6 | NE | 0.399 |
| Df(3L)ED4486 | 69C4;69F6 | NE | 0.461 |
| Df(3L)BSC12 | 69F6--70A1;70A1--2 | En | 1.488 |
| Df(3L)ED4502 | 70A3;70C10 | NE | 0.050 |
| Df(3L)ED4543 | 70C6;70F4 | NE | 0.887 |
| Df(3L)ED217 | 70F4;71E1 | NE | -0.216 |
| Df(3L)BSC845 | 71D3;72A1 | NE | 0.129 |
| Df(3L)BSC774 | 71F1;72D10 | NE | -0.325 |
| Df(3L)ED4606 | 72D4;73C4 | En | 1.597 |
| Df(3L)ED4674 | 73B5;73E5 | NE | -0.156 |
| Df(3L)ED4685 | 73D5;74E2 | NE | 0.299 |
| Df(3L)ED4710 | 74D1;75B11 | NE | 0.430 |
| Df(3L)BSC775 | 75A2;75E4 | NE | 0.065 |
| Df(3L)BSC220 | 75F1;76A1 | NE | 0.273 |
| Df(3L)ED229 | 76A1;76E1 | NE | 0.729 |
| Df(3L)BSC20 | 76A7--B1;76B4--5 | NE | 0.042 |
| Df(3L)ED4858 | 76D3;77C1 | En | 3.402 |
| Df(3L)BSC839 | 77B4;77C6 | NE | -0.501 |
| Df(3L)BSC797 | 77C3;78A1 | En | 4.655 |
| Df(3L)BSC449 | 77F2;78C2 | NE | -0.097 |
| Df(3L)BSC553 | 78A2;78C2 | Su | -1.189 |
| Df(3L)BSC419 | 78C2;78D8 | Su | -1.222 |
| Df(3L)ED4978 | 78D5;79A2 | NE | 0.133 |
| Df(3L)BSC223 | 79A3;79B3 | NE | -0.405 |
| Df(3L)BSC451 | 79B2;79F5 | NE | 0.739 |
| Df(3L)ED230 | 79C2;80A4 | En | 1.150 |
| Df(3L)ED5017 | 80A4;80C2 | NE | 0.522 |
| Df(3L)1-16 | 80F;80F | NE | 0.150 |

| *hop^Tum-l^* 3R Deficiency Screen Results | | | |
| --- | --- | --- | --- |
| **Df symbol** | **Cytology** | **Interaction** | **Normalized TI** |
| [Df(3R)ED5100](http://flybase.org/reports/FBab0036339.html) | 81F6;82E7 | NE | 0.990 |
| [Df(3R)10-65](http://flybase.org/reports/FBab0002465.html) | 81Fa;81Fa | En | 2.281 |
| [Df(3R)ED5147](http://flybase.org/reports/FBab0036386.html) | 82E7;83A1 | NE | -0.353 |
| [Df(3R)ED5156](http://flybase.org/reports/FBab0036395.html) | 82F8;83A4 | NE | -0.367 |
| [Df(3R)BSC549](http://flybase.org/reports/FBab0045441.html) | 83A6;83B6 | NE | -0.551 |
| [Df(3R)ED5177](http://flybase.org/reports/FBab0036416.html) | 83B4;83B6 | NE | 0.256 |
| [Df(3R)BSC47](http://flybase.org/reports/FBab0037761.html) | 83B7;83D1 | NE | 0.999 |
| [Df(3R)BSC464](http://flybase.org/reports/FBab0045330.html) | 83B7;83E1 | NE | 0.106 |
| [Df(3R)Tpl10](http://flybase.org/reports/FBab0002670.html) | 83C1;84B2 | NE | -0.451 |
| [Df(3R)BSC681](http://flybase.org/reports/FBab0045747.html) | 83E2;83E5 | NE | -0.459 |
| [Df(3R)BSC738](http://flybase.org/reports/FBab0045856.html) | 83E5;84A1 | NE | -0.601 |
| [Df(3R)BSC467](http://flybase.org/reports/FBab0045333.html) | 83F1;84B2 | NE | -0.367 |
| [Df(3R)Antp17](http://flybase.org/reports/FBab0002493.html) | 84A5;84D9 | NE | 0.873 |
| [Df(3R)BSC633](http://flybase.org/reports/FBab0045687.html) | 84B2;84C3 | NE | -0.235 |
| [Df(3R)ED7665](http://flybase.org/reports/FBab0037582.html) | 84B4;84E11 | NE | 0.761 |
| [Df(3R)BSC466](http://flybase.org/reports/FBab0045332.html) | 84E1;85A10 | NE | 0.053 |
| [Df(3R)ED5339](http://flybase.org/reports/FBab0036575.html) | 85D1;85D11 | NE | -0.161 |
| [Df(3R)BSC476](http://flybase.org/reports/FBab0045342.html) | 85D16;85D24 | NE | 0.596 |
| [Df(3R)Exel6264](http://flybase.org/reports/FBab0038280.html) | 85D24;85E5 | NE | -0.134 |
| [Df(3R)BSC507](http://flybase.org/reports/FBab0045323.html) | 85D6;85D15 | NE | -0.106 |
| [Df(3R)ED5428](http://flybase.org/reports/FBab0036664.html) | 85E1;85F8 | NE | -0.249 |
| [Df(3R)Exel6154](http://flybase.org/reports/FBab0038209.html) | 85E9;85F1 | NE | -0.246 |
| [Df(3R)Exel6155](http://flybase.org/reports/FBab0038210.html) | 85F1;85F10 | NE | -0.592 |
| [Df(3R)ED5474](http://flybase.org/reports/FBab0036710.html) | 85F11;86B1 | En | 1.197 |
| [Df(3R)BSC621](http://flybase.org/reports/FBab0045562.html) | 85F5;85F14 | NE | -0.131 |
| [Df(3R)BSC568](http://flybase.org/reports/FBab0045461.html) | 86C7;86D7 | NE | -0.071 |
| [Df(3R)BSC469](http://flybase.org/reports/FBab0045335.html) | 86D8;87A2 | NE | -0.678 |
| [Df(3R)ED5577](http://flybase.org/reports/FBab0036807.html) | 86F9;87B13 | NE | -0.180 |
| [Df(3R)BSC486](http://flybase.org/reports/FBab0045302.html) | 87B10;87E9 | NE | 0.067 |
| Df(3R)ED5623 | 87E3;88A4 | En | 1.377 |
| [Df(3R)ED5644](http://flybase.org/reports/FBab0036873.html) | 88A4;88C9 | NE | -0.787 |
| [Df(3R)ED10555](http://flybase.org/reports/FBab0044101.html) | 88C9;88D8 | NE | -0.369 |
| [Df(3R)ED5664](http://flybase.org/reports/FBab0036893.html) | 88D1;88E3 | NE | 0.447 |
| [Df(3R)ED5705](http://flybase.org/reports/FBab0036934.html) | 88E12;89A5 | NE | -0.685 |
| [Df(3R)BSC750](http://flybase.org/reports/FBab0045816.html) | 88E2;88E5 | NE | -0.249 |
| [Df(3R)BSC741](http://flybase.org/reports/FBab0045808.html) | 88E8;88F1 | NE | -0.534 |
| [Df(3R)BSC515](http://flybase.org/reports/FBab0045361.html) | 88F6;89A8 | NE | -0.450 |
| [Df(3R)Exel7328](http://flybase.org/reports/FBab0038316.html) | 89A12;89B6 | NE | 0.052 |
| [Df(3R)BSC728](http://flybase.org/reports/FBab0045797.html) | 89A8;89B2 | NE | -0.345 |
| [Df(3R)ED10642](http://flybase.org/reports/FBab0044185.html) | 89B17;89D5 | NE | -0.047 |
| [Df(3R)Exel6270](http://flybase.org/reports/FBab0038286.html) | 89B18;89D8 | NE | -0.528 |
| [Df(3R)BSC887](http://flybase.org/reports/FBab0046625.html) | 89B6;89B16 | NE | 0.255 |
| [Df(3R)ED10639](http://flybase.org/reports/FBab0044183.html) | 89B7;89B18 | NE | 0.255 |
| [Df(3R)P115](http://flybase.org/reports/FBab0009957.html) | 89B7--8;89E7 | NE | 1.022 |
| [Df(3R)ED5780](http://flybase.org/reports/FBab0037009.html) | 89E11;90C1 | NE | -0.202 |
| [Df(3R)BSC748](http://flybase.org/reports/FBab0045814.html) | 89E5;89E11 | NE | -0.623 |
| [Df(3R)BSC790](http://flybase.org/reports/FBab0045879.html) | 90B6;90E2 | NE | -0.153 |
| [Df(3R)BSC650](http://flybase.org/reports/FBab0045706.html) | 90C6;91A2 | NE | -0.351 |
| [Df(3R)ED5815](http://flybase.org/reports/FBab0037044.html) | 90F4;91B8 | NE | 0.549 |
| [Df(3R)ED2](http://flybase.org/reports/FBab0029731.html) | 91A5;91F1 | NE | 0.414 |
| [Df(3R)ED5938](http://flybase.org/reports/FBab0037159.html) | 91D4;92A11 | NE | -0.007 |
| [Df(3R)ED6025](http://flybase.org/reports/FBab0037246.html) | 92A11;92E2 | NE | -0.395 |
| [Df(3R)BSC517](http://flybase.org/reports/FBab0045363.html) | 92C1;92F13 | NE | -0.420 |
| [Df(3R)BSC141](http://flybase.org/reports/FBab0044884.html) | 92F2;93A1 | NE | -0.274 |
| [Df(3R)BSC43](http://flybase.org/reports/FBab0037760.html) | 92F7;93B6 | NE | -0.507 |
| [Df(3R)BSC819](http://flybase.org/reports/FBab0045931.html) | 93A2;93B8 | NE | 0.741 |
| [Df(3R)Exel6272](http://flybase.org/reports/FBab0038288.html) | 93A4;93B13 | NE | -0.334 |
| [Df(3R)ED10845](http://flybase.org/reports/FBab0044383.html) | 93B9;93D4 | NE | -0.061 |
| [Df(3R)BSC677](http://flybase.org/reports/FBab0045743.html) | 93D1;93F14 | NE | -0.991 |
| [Df(3R)ED6085](http://flybase.org/reports/FBab0037306.html) | 93F14;94B5 | NE | -0.215 |
| [Df(3R)ED6096](http://flybase.org/reports/FBab0037317.html) | 94B5;94E7 | NE | -0.277 |
| [Df(3R)BSC619](http://flybase.org/reports/FBab0045577.html) | 94D10;94E13 | NE | -0.191 |
| [Df(3R)slo3](http://flybase.org/reports/FBab0044803.html) | 94D4;96A18 | NE | -0.394 |
| [Df(3R)BSC137](http://flybase.org/reports/FBab0044878.html) | 94F1;95A4 | NE | -0.502 |
| [Df(3R)BSC489](http://flybase.org/reports/FBab0045305.html) | 94F3;95D1 | NE | 0.023 |
| [Df(3R)Exel6196](http://flybase.org/reports/FBab0038251.html) | 95C12;95D8 | NE | -0.469 |
| [Df(3R)ED6187](http://flybase.org/reports/FBab0037407.html) | 95D10;96A7 | NE | 0.147 |
| [Df(3R)Exel6197](http://flybase.org/reports/FBab0038252.html) | 95D8;95E1 | En | 2.267 |
| [Df(3R)ED6220](http://flybase.org/reports/FBab0037440.html) | 96A7;96C3 | NE | 0.892 |
| [Df(3R)BSC461](http://flybase.org/reports/FBab0045283.html) | 96B15;96D1 | NE | 0.901 |
| [Df(3R)Exel6201](http://flybase.org/reports/FBab0038256.html) | 96C2;96C4 | NE | -0.412 |
| [Df(3R)FDD-0317950](http://flybase.org/reports/FBab0045881.html) | 96C8;96D1 | NE | -0.515 |
| [Df(3R)Exel6202](http://flybase.org/reports/FBab0038257.html) | 96D1;96D1 | NE | 0.097 |
| [Df(3R)Exel6203](http://flybase.org/reports/FBab0038258.html) | 96E2;96E6 | NE | -0.139 |
| [Df(3R)BSC321](http://flybase.org/reports/FBab0045290.html) | 96E6;96E9 | NE | -0.143 |
| [Df(3R)BSC140](http://flybase.org/reports/FBab0044858.html) | 96F1;96F10 | NE | -0.079 |
| [Df(3R)ED6232](http://flybase.org/reports/FBab0037452.html) | 96F10;97D2 | NE | -0.152 |
| [Df(3R)ED6255](http://flybase.org/reports/FBab0037475.html) | 97D2;97F1 | NE | -0.850 |
| [Df(3R)BSC497](http://flybase.org/reports/FBab0045313.html) | 97E6;98B5 | NE | -0.004 |
| [Df(3R)ED6280](http://flybase.org/reports/FBab0037500.html) | 98B6;98B6 | NE | -0.258 |
| [Df(3R)BSC567](http://flybase.org/reports/FBab0045460.html) | 98B6;98E5 | NE | 0.354 |
| [Df(3R)BSC874](http://flybase.org/reports/FBab0046242.html) | 98E1;99A1 | NE | 0.393 |
| [Df(3R)BSC501](http://flybase.org/reports/FBab0045317.html) | 98F10;99B9 | NE | -0.071 |
| [Df(3R)BSC547](http://flybase.org/reports/FBab0045439.html) | 99B5;99C2 | NE | -0.289 |
| [Df(3R)L127](http://flybase.org/reports/FBab0023951.html) | 99B5;99F1 | NE | -1.165 |
| [Df(3R)BSC620](http://flybase.org/reports/FBab0045578.html) | 99C5;99D3 | NE | -0.307 |
| [Df(3R)X3F](http://flybase.org/reports/FBab0002698.html) | 99D1;99E1 | NE | 0.192 |
| [Df(3R)BSC502](http://flybase.org/reports/FBab0045318.html) | 99D3;99D8 | NE | 0.120 |
| [Df(3R)Exel6214](http://flybase.org/reports/FBab0038269.html) | 99D5;99E2 | NE | 0.313 |
| [Df(3R)R133](http://flybase.org/reports/FBab0009005.html) | 99E1--5;3Rt | NE | -0.892 |
| [Df(3R)BSC503](http://flybase.org/reports/FBab0045319.html) | 99E3;99F6 | NE | 0.462 |
| [Df(3R)BSC504](http://flybase.org/reports/FBab0045320.html) | 99F4;100A2 | Su | -1.193 |
| [Df(3R)Exel7378](http://flybase.org/reports/FBab0038321.html) | 99F8;100A5 | NE | 0.494 |
| [Df(3R)A113](http://flybase.org/reports/FBab0002482.html) | 100A;3Rt | NE | -0.628 |
| [Df(3R)ED6346](http://flybase.org/reports/FBab0037564.html) | 100A5;100B1 | En | 5.775 |
| [Df(3R)BSC749](http://flybase.org/reports/FBab0045815.html) | 100B1;100C1 | NE | -0.633 |
| [Df(3R)BSC793](http://flybase.org/reports/FBab0045885.html) | 100B5;100C4 | NE | 0.397 |
| [Df(3R)ED6361](http://flybase.org/reports/FBab0037579.html) | 100C7;100E3 | NE | -0.324 |
| [Df(3R)ED50003](http://flybase.org/reports/FBab0045682.html) | 100E1;100F5 | NE | 0.043 |
