# Supplementary material for: A Genetic Screen Reveals an Unexpected Role for Yorkie Signaling in JAK/STAT-Dependent Hematopoietic Malignancies in Drosophila melanogaster
Source: G3 (Bethesda). 2017 Jun 15;7(8):2427–38. doi: 10.1534/g3.117.044172 (PMC5555452; doi:10.1534/g3.117.044172)
Supplement: Supplementary file 6 [file 2427TableS2.docx]

| **Df modifier**  **(this screen)** | **Df cytology**  **(this screen)** | **Interaction**  **(this screen)** | **Modifier gene/Df name** | **Modifier cytology** | **Reported interaction** | **Citation** |
| --- | --- | --- | --- | --- | --- | --- |
| *Df(2L)ED1203* | 36F7;37C5 | En | *RpL30* | 37B9-37B9 | En of *hop^Tum-l^* | Shi et al., 2006 |
| *Df(3L)BSC117* | 65E9;65F5 | Su | *Df(3L)Exel6111* | 65E7-65F4 | Su of *hop^Tum-l^* | Shi et al., 2006 |
| *Df(3L)BSC816* | 66D9;66D12 | En | *hairy* | [66D10](http://flybase.org/cgi-bin/txtbrowse_fb.html?xfieldname1=CLOC&group=yes&objtype=gene%20cytogene%20tRNA%20ncRNA%20snRNA%20snoRNA%20miRNA%20rRNA%20transposable_element_insertion_site%20cytoins%20deleted_segment%20cytodeleted_segment%20duplicated_segment%20cytoduplicated_segment&xfield1=66D10-66D10) | En of *hop^Tum-l^* | Shi et al., 2006 |
| *Df(3L)ED4858* | 76D3;77C1 | En | *polo* | 77B2-77B3 | En of *hop^Tum-l^* | Shi et al., 2006 |
|  |  |  | *HEM-protein* | 79D4-79D4 | En of *hop^Tum-l^* | Shi et al., 2006 |
| *Df(3R)Exel6197* | 95D8;95E1 | En | *Atg6* | 95D10 | *Atg6* mutants have melanotic tumors | Shravage et al., 2013 |
|  |  |  |  |  |  |  |

**Table S2. Overlap between our screen and genes that affect *hop^Tum-l^* tumors and/or induce melanotic masses.**
